# *Neisseria meningitidis* Translation Elongation Factor P and Its Active-Site Arginine Residue Are Essential for Cell Viability

**DOI:** 10.1371/journal.pone.0147907

**Published:** 2016-02-03

**Authors:** Tatsuo Yanagisawa, Hideyuki Takahashi, Takehiro Suzuki, Akiko Masuda, Naoshi Dohmae, Shigeyuki Yokoyama

**Affiliations:** 1 RIKEN Structural Biology Laboratory, 1-7-22 Suehiro-cho, Tsurumi, Yokohama 230–0045, Japan; 2 RIKEN Systems and Structural Biology Center, 1-7-22 Suehiro-cho, Tsurumi, Yokohama 230–0045, Japan; 3 National Institute of Infectious Disease, Department of Bacteriology, 1-23-1 Toyama, Shinjuku-ku, Tokyo 162–8640, Japan; 4 RIKEN Center for Sustainable Resource Science (CSRS), 2–1 Hirosawa, Wako, Saitama 351–0198, Japan; 5 National Maritime Research Institute, 6-38-1 Shinkawa, Mitaka, Tokyo 181–0004, Japan; University of British Columbia, CANADA

## Abstract

Translation elongation factor P (EF-P), a ubiquitous protein over the entire range of bacterial species, rescues ribosomal stalling at consecutive prolines in proteins. In *Escherichia coli* and *Salmonella enterica*, the post-translational β-lysyl modification of Lys34 of EF-P is important for the EF-P activity. The β-lysyl EF-P modification pathway is conserved among only 26–28% of bacteria. Recently, it was found that the *Shewanella oneidensis* and *Pseudomonas aeruginosa* EF-P proteins, containing an Arg residue at position 32, are modified with rhamnose, which is a novel post-translational modification. In these bacteria, EF-P and its Arg modification are both dispensable for cell viability, similar to the *E*. *coli* and *S*. *enterica* EF-P proteins and their Lys34 modification. However, in the present study, we found that EF-P and Arg32 are essential for the viability of the human pathogen, *Neisseria meningitidis*. We therefore analyzed the modification of Arg32 in the *N*. *meningitidis* EF-P protein, and identified the same rhamnosyl modification as in the *S*. *oneidensis* and *P*. *aeruginosa* EF-P proteins. *N*. *meningitidis* also has the orthologue of the rhamnosyl modification enzyme (EarP) from *S*. *oneidensis* and *P*. *aeruginosa*. Therefore, EarP should be a promising target for antibacterial drug development specifically against *N*. *meningitidis*. The pair of genes encoding *N*. *meningitidis* EF-P and EarP suppressed the slow-growth phenotype of the EF-P-deficient mutant of *E*. *coli*, indicating that the activity of *N*. *meningitidis* rhamnosyl–EF-P for rescuing the stalled ribosomes at proline stretches is similar to that of *E*. *coli* β-lysyl–EF-P. The possible reasons for the unique requirement of rhamnosyl–EF-P for *N*. *meningitidis* cells are that more proline stretch-containing proteins are essential and/or the basal ribosomal activity to synthesize proline stretch-containing proteins in the absence of EF-P is lower in this bacterium than in others.

## Introduction

The ribosome connects amino acids together to synthesize a protein in the order specified by the mRNA sequence. During this translation process, multiple proline stretches with two or more consecutive prolines in the amino acid sequence retard peptide bond formation [1] and cause ribosome stalling [2]. Translation elongation factor P (EF-P) alleviates ribosome stalling at proline stretches [3, 4, 5, 6, 7, 8, 9, 10], by binding between the peptidyl (P) site and the tRNA exit (E) site of the ribosome [11, 12]. EF-P was discovered as a protein that stimulates the ribosomal peptidyltransferase activity [13, 14, 15], and is almost universally conserved among bacteria [16].

In the EF-P proteins from *Escherichia coli* and *Salmonella enterica*, the lysine residue at position 34 is post-translationally modified to (*R*)-β-lysyl(γ or δ-hydroxy)lysine. For this modification, first the lysine 2,3-aminomutase EpmB (also called YjeK) forms (*R*)-β-lysine from L-lysine. EpmA (also called PoxA, YjeA, and GenX) then attaches the (*R*)-β-lysine to the ε-amino group of Lys34 [16, 17, 18, 19]. Finally, the β-lysyllysine hydroxylase EpmC (also called YfcM) hydroxylates the Lys34 side chain [20, 21, 22] to complete the modification. The β-lysyl modification, but not the hydroxylation, of Lys34 is crucial for the EF-P activity to alleviate ribosome stalling [21, 23]. Lack of the β-lysyl modification of EF-P(Lys34) causes a variety of phenotypes in bacteria, such as alterations in cell growth, pathogenicity, stress response, motility, and resistance to antibiotics and detergents [17, 18, 24, 25, 26].

EF-P is composed of domains 1, 2, and 3, and the overall structure assumes an L shape, which mimics that of tRNA [18, 27, 28]. In contrast, EpmA is a paralogue of lysyl-tRNA synthetase (LysRS). Therefore, the β-lysyl modification of EF-P Lys34 by EpmA may be regarded as molecular mimicry, in that Lys34 corresponds to the 3′-end adenosine (A76) of tRNA, and the mechanisms of substrate recognition and aminoacylation catalysis by EpmA resemble those of an aminoacyl-tRNA synthetase [18, 29]. The (*R*)-β-lysyl group of the post-translationally modified Lys34, at the tip of the L-shaped EF-P, may contact the ribosomal peptidyltransferase center [12].

Lys34 is conserved in the EF-Ps among about 80% of bacteria. However, the β-lysyl modification enzymes EpmA and EpmB are conserved in only 26–28% of bacteria, including *E*. *coli* and its phylogenetically related γ-proteobacteria (*e*.*g*., *Enterobacter aerogenes*, *Salmonella enterica*, *Vibrio cholerae*, *Shigella flexneri*, *Haemophilus influenzae*, and *Yersinia pestis*). Therefore, the other types of post-translational lysine modifications, if any, of the EF-Ps from a large number of bacteria are still not known. In this regard, initiation factor 5A (e/aIF5A) is the eukaryotic/archaeal orthologue of EF-P, and its highly-conserved lysine residue (Lys50) is post-translationally modified to hypusine by deoxyhypusine synthase (DHS) and deoxyhypusine hydroxylase (DOHH) [30, 31, 32]. Moreover, the deoxyhypusine modification is important for translation elongation and restoration of stalled ribosomes [33, 34].

The EF-P proteins from 9–14% of bacteria, including β-proteobacteria (*e*.*g*., *Neisseria meningitidis*, *Neisseria gonorrhoeae*, *Bordetella pertussis*, *Burkholderia cepacia*, *Kingella kingae*, and *Ralstonia solanacearum*), γ-proteobacteria (*e*.*g*., *Pseudomonas aeruginosa*, *Pseudomonas fluorescens*, and *Aeromonas hydrophila*), Spirochaetes (*Borrelia burgdorferi*), and Deinococci (*Thermus thermophilus* and *Deinococcus radiodurans*) [16, 35], have an Arg residue at the position corresponding to Lys34. Recently, it was reported that the *Shewanella oneidensis* and *Pseudomonas aeruginosa* EF-P proteins, containing the Arg residue at position 32, are modified with rhamnose, a novel post-translational modification [35]. The corresponding modification enzymes have been identified, and are considered to be conserved in bacteria with this particular Arg residue in EF-P [35, 36].

In bacteria, including *E*. *coli*, the EF-P deletion is not lethal [35, 36, 37], although the genome encodes over one thousand proline stretches in its proteins. This is probably because even a protein containing a strong pausing consecutive proline sequence, such as PPPP, can be expressed at the basal level: *i*.*e*., at several percent relative to a protein with no proline stretch [9]. Similarly, the deficiency in the enzyme activity for the known Lys or Arg modification is not lethal. Consequently, EF-P and its Lys/Arg modification are important, but not essential, to alleviate ribosome stalling at proline stretches.

In the present study, we studied the EF-P protein from *Neisseria meningitidis*, a member of the β-proteobacteria and a leading cause of bacterial meningitis and septicemia worldwide, which is therefore a potential target for drug development. *N*. *meningitidis* has EF-P containing Arg32 and its putative modification enzyme. Remarkably, the number of proline stretches encoded in the *N*. *meningitidis* genome is much smaller than those in the genomes of other bacteria, including *E*. *coli*, *S*. *oneidensis* and *P*. *aeruginosa*. However, it was unknown whether *N*. *meningitidis* EF-P, or EF-P(*Nm*), is essential for viability, and whether EF-P(*Nm*) is post-translationally modified in the same manner as in *S*. *oneidensis* and *P*. *aeruginosa*.

Therefore, we analyzed the modification of Arg32 in EF-P(*Nm*), and identified it as the same rhamnosylation as those of the *S*. *oneidensis* and *P*. *aeruginosa* EF-P proteins. We successfully deleted the *N*. *meningitidis* gene encoding the EF-P rhamnosyl modification enzyme, EarP. However, our attempt to disrupt the *N*. *meningitidis* gene encoding EF-P failed, indicating that EF-P is essential for cell viability. We confirmed that, in contrast to most bacteria, both EF-P(*Nm*) and Arg32 are crucial for the viability of *N*. *meningitidis*.

## Results and Discussion

### *N*. *meningitidis* EF-P is essential for cell viability

**Fig 1:**
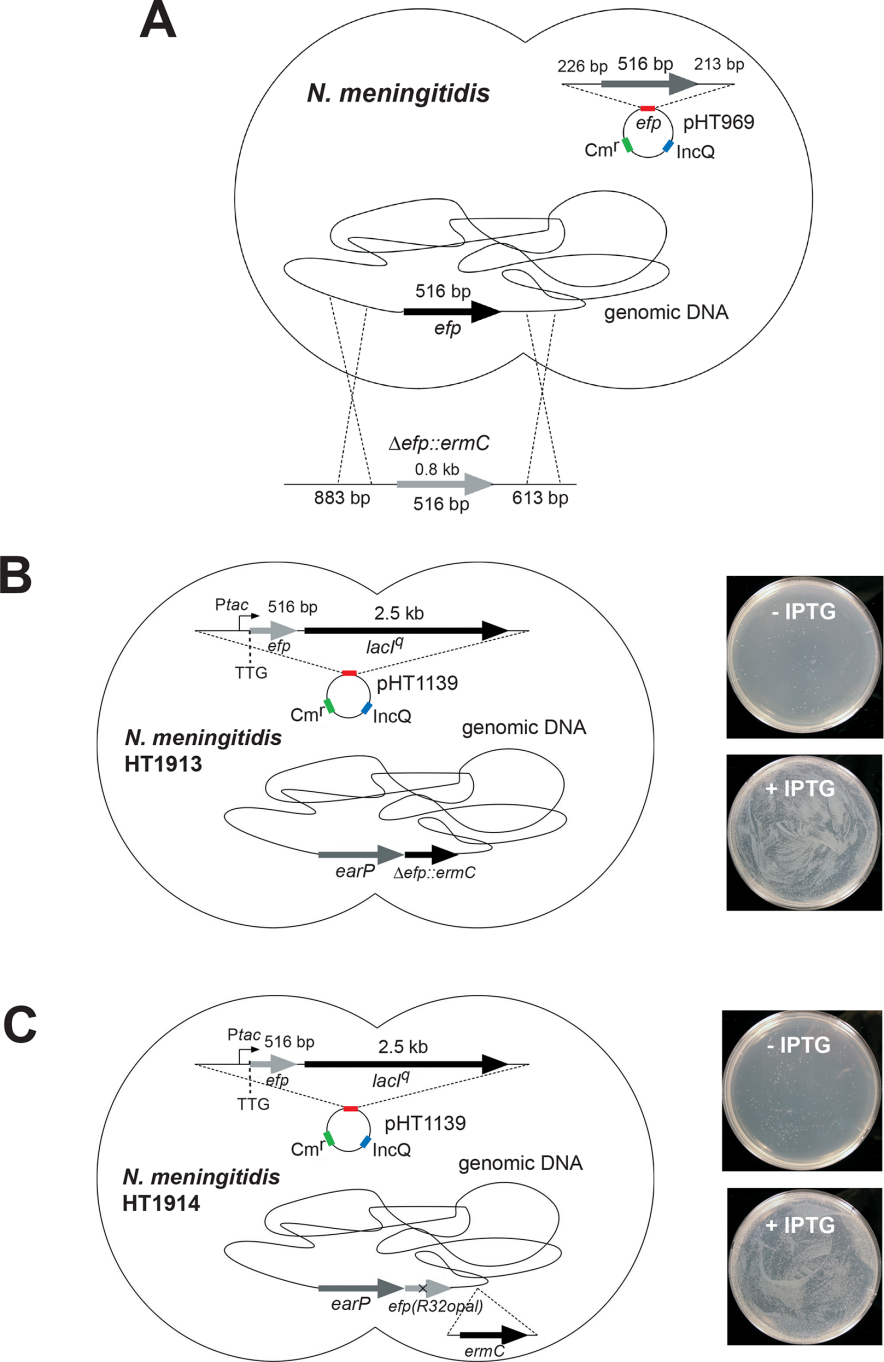
Strategies for *efp* deletion from the *N*. *meningitidis* genome. (A) The *efp* allele in the *N*. *meningitidis* genome can be disrupted, but only in the presence of a plasmid containing the wild-type *N*. *meningitidis efp* gene. (B, C) The IncQ plasmid pHT1139, containing P*tac-*TTG*-efp-lacI*^*q*^, was transformed into *N*. *meningitidis* H44/76 cells. Subsequently, the DNA fragment bearing the erythromycin resistance gene (*ermC*) or the *earP*-*efp*(*R32opal*) gene was introduced into the *efp* allele within the *N*. *meningitidis* H44/76/pHT1139 genome, to obtain the *N*. *meningitidis* strains HT1913/pHT1139 (B, left) and HT1914/pHT1139 (C, left), respectively. In these strains, the *efp* gene expression can be controlled by IPTG, and EF-P can be inducibly produced in the presence of IPTG. (B, C, right) Growth of the *N*. *meningitidis* HT1913/pHT1139 and HT1914/pHT1139 cells, with and without IPTG. Both of the *N*. *meningitidis* cells lack the *efp* gene in the genome, but contain an inducible copy of the *efp* gene in the IncQ plasmid.

We first tried to disrupt the *efp* gene, encoding EF-P, in the *N*. *meningitidis* genome, but could not obtain any erythromycin-resistant (Erm^r^) colonies with the *Δefp*::*ermC* allele (data not shown). This result suggested that the *efp* gene is essential for *N*. *meningitidis* viability. To further examine this possibility, *N*. *meningitidis* cells with the endogenous *efp* gene in the chromosome were transformed with pHT261 (S1 Table), derived from the broad-host-range IncQ plasmid and harboring a second *efp* gene, which is designated hereafter as pHT969 (Fig 1A). These meningococcal transformants were further transformed with a PCR fragment containing the *efp*-flanking regions and the *ermC* gene, in order to disrupt the *efp* gene in the chromosome. Numerous colonies of the erythromycin-resistant mutant were obtained for *N*. *meningitidis* cells harboring pHT969 (the wild-type *efp*-containing plasmid). In contrast, for *N*. *meningitidis* cells harboring pHT261 (the empty vector plasmid), very few colonies of the erythromycin-resistant mutant(s) were obtained, and they lacked the *ermC* gene in the *efp* locus. These results indicated that the *N*. *meningitidis efp* gene is essential for cell viability (Table 1).

**Table 1:** The *N*. *meningitidis efp* gene is essential for cell viability.

Strain / plasmid (*efp* gene)	No. of *Δefp*::*ermC* transformants
*N*. *meningitidis* HT 1125 / pHT261 [vector]	0
*N*. *meningitidis* HT1125 / pHT969 [wild-type *efp*]	16,919 (+/- 8,532)
*N*. *meningitidis* HT1125 / pHT971 [*efp*(R32K)]	0
*N*. *meningitidis* HT1125 / pHT972 [*efp*(R32A)]	0

A DNA fragment containing the *erm*C gene and the *efp* flanking regions was transformed into *N*. *meningitidis* HT1125, but the disruption of the *efp* gene failed in the absence of the wild-type *efp*-containing plasmid. Experiments were performed 5 times and the averages of the results are shown, together with the standard deviation for the wild-type *efp*, in this table.

In parallel, we performed a complementary experiment to assess whether the *efp* gene is actually essential for *N*. *meningitidis* viability. First, *N*. *meningitidis* cells were transformed with the IncQ plasmid pHT1139 (S1 Table), containing an IPTG-inducible copy of the *efp* gene under the control of the *tac* promoter. Then, under conditions with the induced expression of the *efp* gene, we deleted the *efp* gene from the *N*. *meningitidis* H44/76 genome, by integrating an erythromycin resistance gene (*ermC*) or the *efp* gene with the Arg32 codon replaced by an opal (TGA) stop codon. The growth characteristics of the *N*. *meningitidis* cells containing the inducible *efp* gene, with and without the inducer, are shown in Fig 1B and 1C, respectively. Without IPTG, the *N*. *meningitidis* HT1913/pHT1139 and HT1914/pHT1139 cells barely grew, and the very small number of colonies should be ascribed to the leaky expression of the *efp* gene in the *N*. *meningitidis* cells grown in the absence of IPTG. In contrast, IPTG restored the growth of both *N*. *meningitidis* cells, and large numbers of colonies were observed (Fig 1B and 1C, right). Consequently, *N*. *meningitidis efp* is essential for cell viability.

In addition, meningococcal transformants with a plasmid harboring the *efp*(R32K) or *efp*(R32A) mutant, with Arg32 replaced by either Lys32 or Ala32, respectively, were also constructed. As a result, almost no colonies of the *efp*-null mutant were obtained in the presence of the plasmid harboring the *efp*(R32K) or *efp*(R32A) mutant gene (Table 1). Thus Arg32 is indispensable for the EF-P activity in *N*. *meningitidis*. Conversely, the *efp* gene disruption is not lethal in other bacteria, such as *E*. *coli* MG1655 [37], *E*. *coli* W3110 [38], *S*. *enterica* [17], *Agrobacterium tumefaciens* [24], *P*. *aeruginosa* [35, 36, 39], and *Bacillus subtilis* [40]. This is the first report that the EF-P function is essential for cell viability.

### *N*. *meningitidis* EF-P is post-translationally modified at Arg32

**Fig 2:**
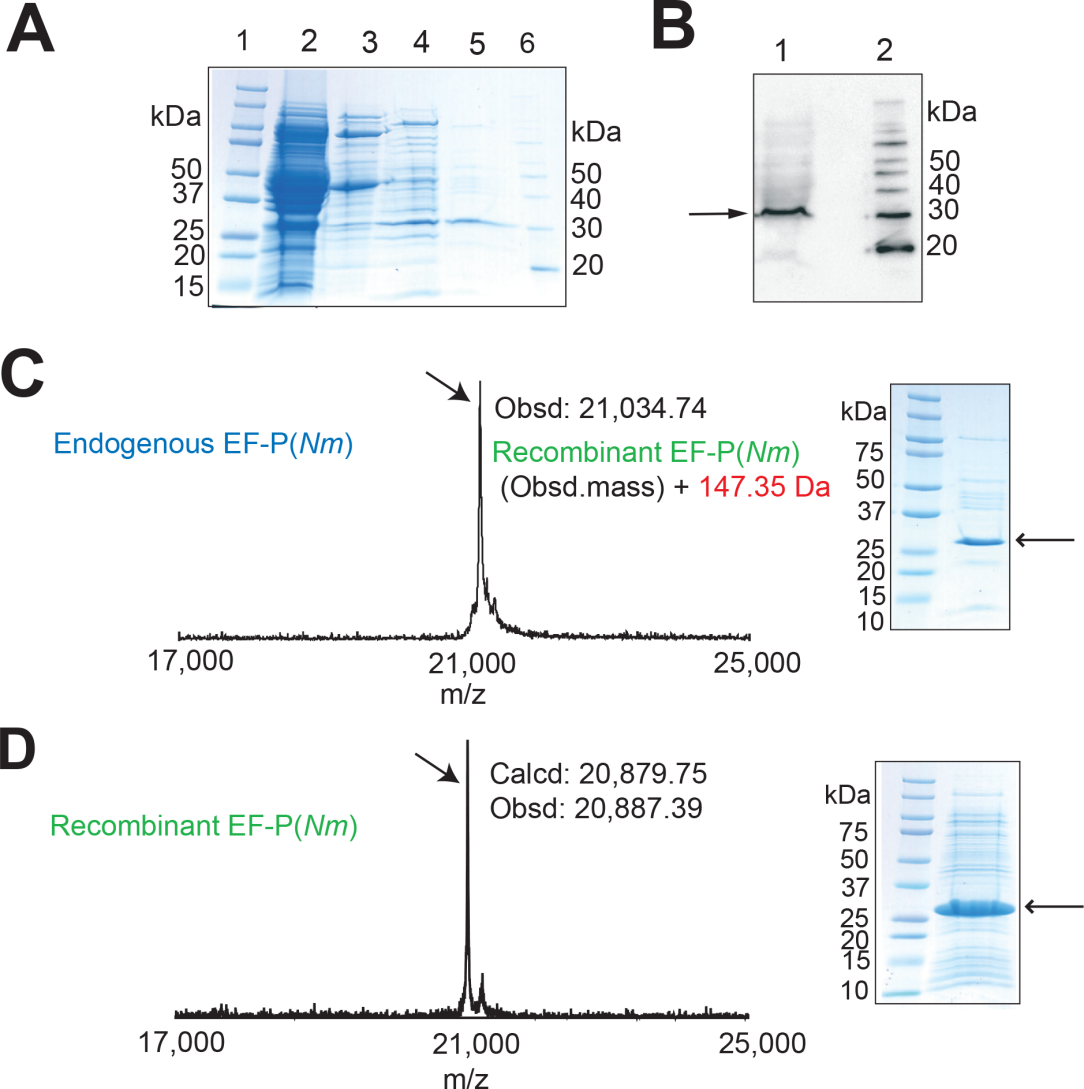
Purification and MS analysis of *N*. *meningitidis* EF-P(*Nm)*. Proteins in each purification step were analyzed by 10–20% SDS-PAGE and stained with SimplyBlue SafeStain. (A) Lane 1, molecular mass standards; lane 2, *N*. *meningitidis* crude cell extract; lane 3, after DEAE-Sephacel column; lane 4, after HiTrap Q HP column; lane 5, endogenous EF-P(*Nm*) purified on a HiTrap Butyl HP column; lane 6, MagicMark molecular mass standards (Life Technologies). (B) The polyclonal antibody against EF-P(*Ec*) cross-reacts with EF-P(*Nm*). The EF-P proteins in the *N*. *meningitidis* cell extracts and the column chromatography fractions were monitored by western blotting with the antibody. Lane 1, *N*. *meningitidis* crude cell extract; lane 2, MagicMark molecular mass standards. The arrow designates EF-P(*Nm*). (C) MALDI-TOF MS and SDS-PAGE of the endogenous EF-P(*Nm*), shown in the left and right panels, respectively. (D) MALDI-TOF MS and SDS-PAGE of the recombinant EF-P(*Nm*), shown in the left and right panels, respectively.

**Fig 3:**
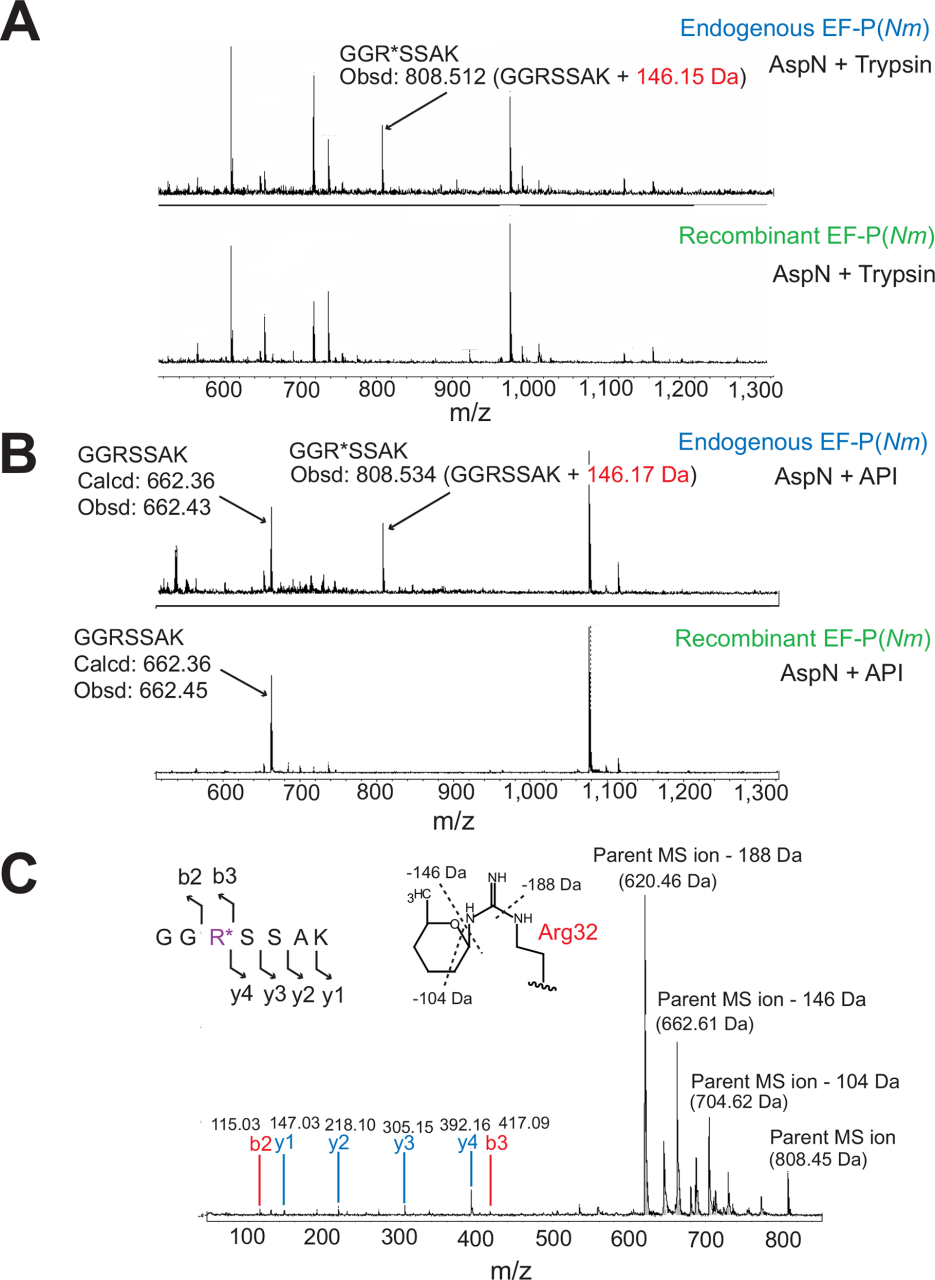
PMF and MS/MS analyses of *N*. *meningitidis* endogenous EF-P. (A, B) The endogenous and recombinant EF-P(*Nm*) proteins were digested with AspN and trypsin (A) or with AspN and API (B), and were subjected to the PMF analysis. The peptide “GGR*SSAK” (R* represents modified Arg32) of the endogenous EF-P(*Nm*) was larger by 146 Da (obsd: 808.512 [M+H]^+^ and 808.534 [M+H]^+^), than the calculated mass of the peptide “GGRSSAK” obtained from the recombinant EF-P(*Nm*) (calcd: 662.36 [M+H]^+^, obsd: 662.43 [M+H]^+^). (C) MS/MS spectrum of the AspN and trypsin-digested endogenous EF-P(*Nm*). The sequence can be read from the annotated b (blue) or y (red) ion series; the b2, b3, y1, y2, y3, and y4 ions were observed. The parent ion (obsd: 808.454 [M+H]^+^), and parent ion –104 Da (obsd: 704.624 [M+H]^+^), parent ion –146 Da (obsd: 662.61 [M+H]^+^), and parent ion –188 Da (obsd: 620 462 [M+H]^+^) generated by the degradation of the modified arginine residue were also observed. Possible degradation sites of the modified arginine are represented by dotted lines.

With the finding that EF-P is essential for *N*. *meningitidis* viability, we next attempted to examine whether there is any difference in the post-translational modification of the EF-P proteins between *N*. *meningitidis* and *S*. *oneidensis*/*P*. *aeruginosa*. First, we analyzed whether EF-P(*Nm*) is post-translationally modified. As the *N*. *meningitidis* cells can only be cultured on solid media, we prepared 100 plates to harvest a sufficient amount of the *N*. *meningitidis* HT1125 cells. The endogenous EF-P was purified from the cells in three column chromatography steps, as described in the “Materials and methods” (Fig 2A and S1 Fig). The molecular mass of the endogenous EF-P(*Nm*) was estimated to be 30 kDa by SDS-PAGE (Fig 2A and 2B), and was slightly larger than that of the EF-P from *E*. *coli* (EF-P(*Ec*)) [18, 41]. MALDI-TOF MS and ESI-MS analyses indicated that the molecular masses of the endogenous EF-P(*Nm*) were 21,034.74 Da (Fig 2C) and 21,040.00 Da (data not shown), respectively, which are higher by 147–153 Da than that of the recombinant EF-P(*Nm*) (obsd: 20,887.39, calcd: 20,879.75) (Fig 2D, S2 Table). A peptide mass fingerprinting (PMF) analysis of the endogenous EF-P(*Nm*) was performed to identify the peptide segment with the post-translational modification (Fig 3A and 3B). The endogenous EF-P(*Nm*) generated peptides with the molecular masses of 808.51 Da (with AspN and trypsin digestion) and 808.53 Da (with AspN and API digestion), which are +146 Da higher than that of the recombinant EF-P(*Nm*) peptide “GGRSSAK” (calcd: 662.36 [M+H]^+^, obsd: 662.45 [M+H]^+^) (Fig 3B). The endogenous EF-P(*Nm*) peptide was further investigated by MALDI-TOF MS/MS analysis (Fig 3C), which confirmed that Arg32 is modified with a +146 Da moiety. Therefore, the post-translational modification of EF-P(*Nm*) occurs at the same position as Arg32 in the *S*. *oneidensis* and *P*. *aeruginosa* EF-P proteins and Lys34 in EF-P(*Ec*) (S2 Fig).

### The post-translational modification at Arg32 of *N*. *meningitidis* EF-P is rhamnosylation

**Fig 4:**
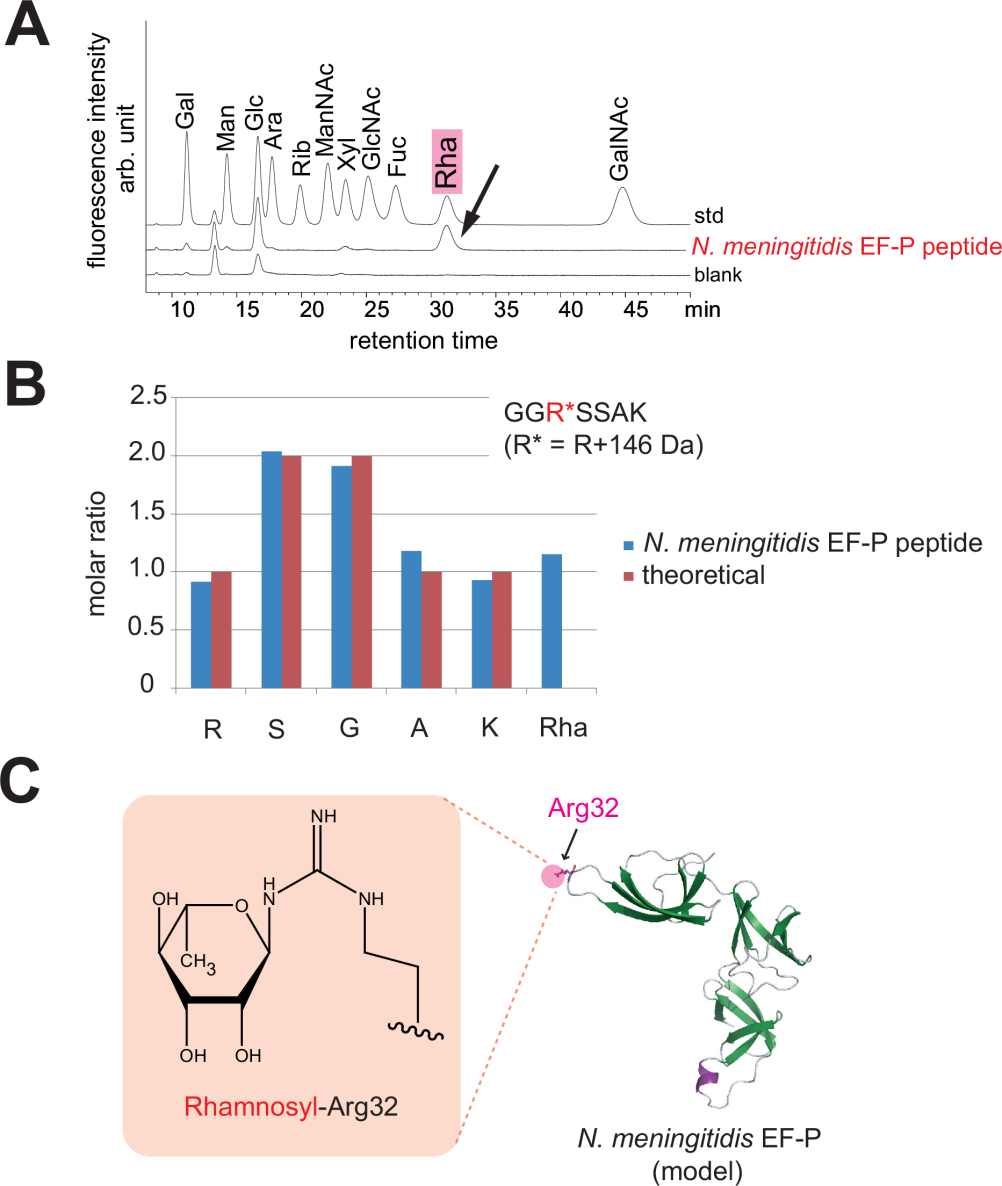
Analysis of the sugar and amino acid components in the modified peptide of *N*. *meningitidis* endogenous EF-P. (A) Analysis of the sugar composition in the endogenous EF-P(*Nm*) peptide, GGR*SSAK. The peptide was hydrolyzed to free amino acids and other components. The samples were derivatized with ABEE, and were subjected to the UHPLC analysis, using 11 monosaccharides as standards (std). A blank sample including deionized water was also loaded (blank). Gal, galactose; Man, mannose; Glc, glucose; Ara, arabinose; Rib, ribose; ManNAc, *N*-acetylmannosamine; Xyl, xylose; GlcNAc, *N*-acetylglucosamine; Fuc, fucose; Rha, rhamnose; GalNAc, *N*-acetylgalactosamine. (B) Quantitative analysis of the amino acid and rhamnose components in the EF-P(*Nm*) peptide. (C) Rhamnosyl–Arg32 and a 3D structural model of *N*. *meningitidis* EF-P.

Arg32 was apparently modified with a monosaccharide, because the modified arginine decomposed during MALDI-TOF MS using α-cyano-4-hydroxycinnamic acid (CHCA) as the matrix. The +146 Da monosaccharide may be either a rhamnosyl or fucosyl modification [35]. *S*. *oneidensis* and *P*. *aeruginosa* only have the biosynthesis pathway for dTDP-rhamnose, and lack that for GDP-fucose, and their EF-P proteins are ramnosylated [35, 36]. However, *N*. *meningitidis* might have the fucose pathway in addition to the rhamnose pathway, as a few of its genes appear to be homologous (E values of 3e–12 and 4e–5 by the Protein BLAST, 40) to the *fcl* gene, encoding the key fucose pathway enzyme in *E*. *coli*. Therefore, we performed an HPLC analysis using 11 monosaccharides as standards, and found that the +146 Da modification is rhamnose (Fig 4A). Furthermore, a quantitative analysis of the sugar and amino acid components revealed that the endogenous EF-P(*Nm*) peptide contains Arg, Ser, Gly, Ala, Lys, and rhamnose, at concentration ratios of 1: 2: 2: 1: 1: 1, respectively (Fig 4B). Taken together, these data demonstrated that the guanidino group of Arg32 in EF-P(*Nm*) is linked with rhamnose in the endogenous EF-P(*Nm*) (Fig 4C), which is the same post-translational modification as in the *S*. *oneidensis* and *P*. *aeruginosa* EF-P proteins [35].

### The *N*. *meningitidis* HT1125 genome encodes the EF-P rhamnosylation enzyme EarP

We found that EF-P(*Nm*) can be modified with rhamnose by crude extracts of *N*. *meningitidis* cells (S3 Fig). In the cases of the *S*. *oneidensis* and *P*. *aeruginosa* EF-P proteins, EarP performs the rhamnosyl modification at Arg32, using dTDP-L-rhamnose as a substrate [35, 36]. The *earP* gene encoding EarP (NMB0935a, accession code: YP_008920709; NMH_0797, accession code: EFV64284) is located next to the *efp* gene (NMB0937, accession code: AAF41343; NMH_0798, accession code: EFV64285) in the genomes of *N*. *meningitidis* strains MC58 and H44/76, while the genome sequence of the *N*. *meningitidis* HT1125 strain is not yet available. The gene encoding EarP was cloned from *N*. *meningitidis* HT1125, and it shared 96% amino acid sequence identity with those from the H44/76 and MC58 strains (S4 Fig).

### The EarP protein from *N*. *meningitidis* modifies EF-P(*Nm*) with rhamnose

**Fig 5:**
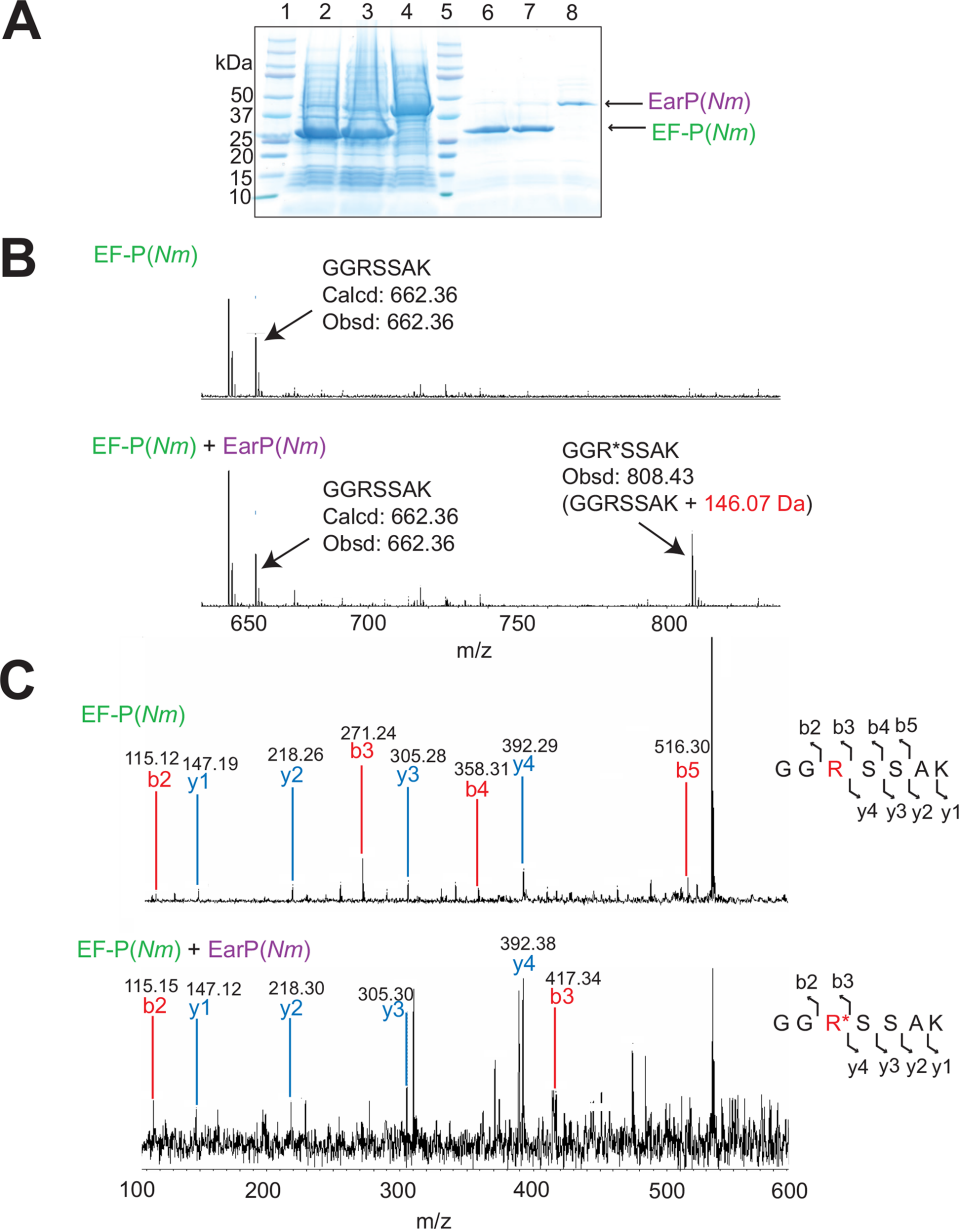
Rhamnosyl modification of the recombinant EF-P(*Nm*) by EarP(*Nm*). (A) Coexpression of EF-P(*Nm*) with EarP(*Nm*) in *E*. *coli* cells. Lane 1, molecular mass standards; lane 2, crude extract of *E*. *coli* cells producing EF-P(*Nm*); lane 3, crude extract of *E*. *coli* cells producing EF-P(*Nm*) and EarP(*Nm*); lane 4, crude extract of *E*. *coli* cells producing EarP(*Nm*); lane 5, molecular mass standards; lane 6, the recombinant EF-P(*Nm*) purified from the cells producing EF-P(*Nm*); lane 7, the recombinant EF-P(*Nm*) purified from the cells producing EF-P(*Nm*) and EarP(*Nm*); lane 8, purified EarP(*Nm*). (B) PMF analysis of the modified and unmodified EF-P(*Nm*). The recombinant EF-P(*Nm*) proteins were purified from the cells producing EF-P(*Nm*), with or without EarP(*Nm*). After digestion with AspN and API, the PMF analysis of the peptides was performed. (C) MS/MS analyses of the recombinant EF-P(*Nm*) and the recombinant EF-P(*Nm*) modified with EarP(*Nm*). After digestion with AspN and API, the EF-P(*Nm*) peptides with masses of 662.35 Da (GGRSSAK) and 808.4 Da (GGR*SSAK, R* designates the modified Arg32) were subjected to the MS/MS analysis. The sequence can be read from the annotated b (blue) or y (red) ion series; the b2, b3, b4, b5, y1, y2, y3, and y4 ions from the peptide “GGRSSAK” and the b2, b3, y1, y2, y3, and y4 ions from the peptide “GGR*SSAK” were observed.

We tested whether the *N*. *meningitidis* EarP, or EarP(*Nm*), could rhamnosylate EF-P(*Nm*) (Fig 5). The plasmid pET-*Nm*ED was constructed to express EF-P(*Nm*) and EarP(*Nm*) (S1 Table), and EF-P(*Nm*) was coexpressed with EarP(*Nm*) in *E*. *coli* cells (Fig 5A). The MALDI-TOF MS analysis revealed that the observed molecular mass of the recombinant EF-P(*Nm*) (calcd: 21,307.07, obsd: 21,310.12), purified from the cells producing EF-P(*Nm*) and EarP(*Nm*), is 148.4 Da larger than that of the recombinant EF-P(*Nm*) (calcd: 21,161.02, obsd: 21,161.73), purified from the cells producing only EF-P(*Nm*) (S5 Fig, S2 Table). The PMF analysis confirmed that the observed mass of the endogenous EF-P(*Nm*) peptide (808.43 [M+H]^+^) is 146.07 Da larger than the calculated mass of the recombinant EF-P(*Nm*) peptide (calcd: 662.36 [M+H]^+^, obsd: 662.36 [M+H]^+^) (Fig 5B). Furthermore, the MS/MS analysis demonstrated that Arg32 of EF-P(*Nm*) is modified with rhamnose by EarP(*Nm*), not only in *N*. *meningitidis* cells but also in *E*. *coli* cells (Fig 5C), as described in the previous study of *S*. *oneidensis* [35]. The incubation of EF-P(*Nm*) with the *N*. *meningitidis* crude extract resulted in its rhamnosyl modification, indicating that the *N*. *meningitidis* cells contain the precursor substrate, dTDP-rhamnose (S3 Fig).

### The rhamnosylated EF-P(*Nm*), but not the unmodified EF-P(*Nm*), restores the growth rate of EF-P-deleted *E*. *coli* cells to the wild-type level

To examine whether the rhamnosylated EF-P(*Nm*) functions in *E*. *coli* cells, we used the Keio collection of an *E*. *coli* K12 deletion mutant, the Δ*efp* strain JW4107 (BW25113 Δ*efp*::*kan*). The Δ*efp* mutant grew more slowly than the parent strain BW25113, designated hereafter as the wild type (S6 Fig). As described previously [18], the Δ*efp* mutant cells transformed with the plasmid vector pMW119, harboring the *E*. *coli efp*, *epmA*, and *epmB* genes (pMW-*Ec*EGY), grew as fast as the wild-type cells, while the Δ*efp* mutant cells transformed with the empty vector, pMW119, grew as slowly as the parent Δ*efp* mutant cells (S6 Fig). Interestingly, the plasmid pMW-*Nm*E, containing the *N*. *meningitidis efp* gene, actually slowed the cell growth, as compared with the growth of cells transformed with the empty vector (S6 Fig). In contrast, the Δ*efp* mutant cells transformed with the plasmid pMW-*Nm*ED, containing both the *N*. *meningitidis efp* and *earP* genes, grew as fast as the wild-type cells (S6 Fig). As EF-P(*Nm*) has Arg32 at this position, instead of Lys, EF-P(*Nm*) cannot be β-lysylated by *E*. *coli* EpmA and EpmB. Thus, the rhamnosylated EF-P(*Nm*), rather than its unmodified version, is required for the complementation of the EF-P deficiency of *E*. *coli* cells. Therefore, the unmodified EF-P(*Nm*) is non-functional, but the rhamnosyl–EF-P(*Nm*) functions, in place of the endogenous β-lysyl–EF-P(*Ec*), on the *E*. *coli* ribosome.

### Rhamnosyl–EF-P(*Nm*) rescues ribosomes stalled at proline stretches in proteins

**Fig 6:**
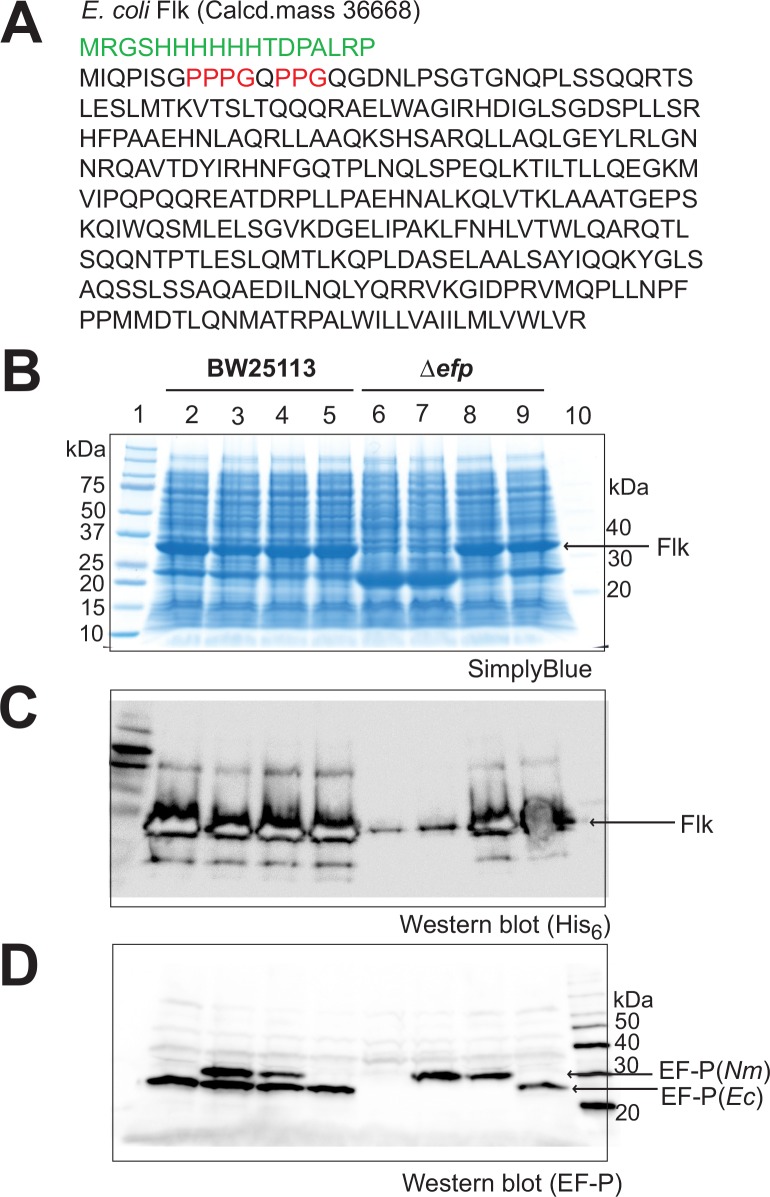
Restoration of production of the *E*. *coli* Flk protein, containing proline stretches, in *efp*-deficient cells. (A) Amino acid sequence of the *E*. *coli* Flk protein. Proline stretches in Flk are colored red. (B) Plasmids pMW119, pMW-*Nm*E (a pMW119-derived plasmid for expressing EF-P(*Nm*)), pMW-*Nm*ED (a pMW119-derived plasmid for expressing EF-P(*Nm*) and EarP(*Nm*)), and pMW-*Ec*EGY (a pMW119-derived plasmid for expressing *E*. *coli* EF-P, EpmA, and EpmB) were cotransformed with the Flk plasmid into *E*. *coli* BW25113 and JW4107 (BW25113 Δ*efp*::*kan*) cells. Whole-cell proteins were subjected to SDS-PAGE, and analyzed for the production of the full-length Flk protein. Lane 1: molecular mass standards; lane 2: BW25113/pMW119; lane 3: BW25113/pMW-*Nm*E; lane 4: BW25113/pMW-*Nm*ED; lane 5: BW25113/pMW-*Ec*EGY; lane 6: JW4107/pMW119; lane 7: JW4107/pMW-*Nm*E; lane 8: JW4107/pMW-*Nm*ED; lane 9: JW4107/pMW-*Ec*EGY; lane 10, MagicMark molecular mass standards. Expression of the chloramphenicol acetyltransferase gene in the Flk plasmid was also observed (23 kDa). (C) The Flk protein was recognized by an anti-His_6_-antibody. (D) Western blotting using a polyclonal antibody against *E*. *coli* EF-P. The expressed EF-P(*Nm*) cross-reacted with the anti-EF-P(*Ec*) antibody.

To examine whether rhamnosyl**–**EF-P(*Nm*) rescues stalled ribosomes at proline stretches in Δ*efp E*. *coli* cells, we used the *E*. *coli* flagellar regulator Flk, consisting of 331 amino acid residues, and *E*. *coli* GntX, consisting of 227 amino acid residues, as model proteins containing proline stretches, such as the PPP and PPG motifs (Fig 6A and S7A Fig). In wild-type *E*. *coli* cells, the full-length Flk and GntX proteins were produced well from the *flk* gene or *gntX* gene-containing plasmid, when co-transformed with any one of the plasmids pMW119, pMW-*Nm*E, pMW-*Nm*ED, and pMW-*Ec*EGY (Fig 6B and 6C and S7B and S7C Fig, lanes 2**–**5). In contrast, in the Δ*efp* cells, the Flk and GntX proteins were only negligibly expressed upon co-transformation with the empty vector pMW119 (Fig 6B and 6C and S7B and S7C Fig, lane 6), probably because the protein synthesis machinery in the Δ*efp* cells stalls at the proline stretches in Flk and GntX. The plasmid pMW-*Nm*E failed to increase the expression of the full-length Flk and GntX proteins in the Δ*efp* cells (Fig 6B and 6C and S7B and S7C Fig, lane 7). In contrast, the plasmid pMW-*Nm*ED completely restored the expression of the full-length Flk and GntX proteins in the Δ*efp* cells to the wild-type level (Fig 6B and 6C and S7B and S7C Fig, lane 8). Therefore, the plasmids pMW-*Nm*ED and pMW-*Ec*EGY caused similar increases in the expression of Flk and GntX in the Δ*efp* cells (Fig 6B and 6C and S7B and S7C Fig, lanes 8 and 9), indicating that the rhamnosyl**–**EF-P(*Nm*) has the same level of activity as that of the β-lysyl**–**EF-P(*Ec*) to rescue *E*. *coli* ribosomes stalled at proline stretches. EF-P(*Nm*) and EarP(*Nm*) can probably fully support the synthesis of proteins with proline stretches in the Δ*efp E*. *coli* cells. The production levels of the recombinant EF-P(*Nm*) protein and the endogenous EF-P(*Ec*) protein in cells were confirmed to be comparable by western blotting (Fig 6D and S7D Fig).

### Proline stretch-containing proteins encoded by the *N*. *meningitidis* genome

We wondered why the EF-P(*Nm*) function is indispensable for cell viability. Approximately 1,500–2,000 proline stretches (XPPX), including 280 strong pausing sequences such as PPPPPP, PPPP, PPP, and PPG, are encoded in the *E*. *coli* genome [3, 4, 5, 6]. Nevertheless, the genes encoding EF-P(*Ec*) and its modification enzymes, EpmA, EpmB, and EpmC, are dispensable for *E*. *coli* viability, although EF-P(*Nm*) and its Arg32 are essential for *N*. *meningitidis* viability. We searched the genome of *N*. *meningitidis* MC58 for proline stretch-containing proteins, and found 850 proline stretches (XPPX), with 77 proteins including 3 PPPP, 45 PPP, and 36 PPG (S3 Table). The *N*. *meningitidis* genome is approximately 2.2 megabase pairs in size with a GC content of 51%, and encodes 2,063 proteins [42], while the *E*. *coli* genome is ca. 4.64 megabase pairs in size with a GC content of about 50.8%, and encodes 4,289 proteins [43]. The similar GC contents of the two genomes suggest that the statistical probabilities of the Pro codons (CCU, CCC, CCA, and CCG) are also similar between them. Thus, the frequency of the genes encoding proline stretch-containing proteins in the *N*. *meningitidis* genome is actually smaller than that in the *E*. *coli* genome, and may not be relevant to the essentiality of the EF-P(*Nm*) function. However, among the 77 *N*. *meningitidis* proline stretch-containing proteins, five of the *E*. *coli* homologues are essential for *E*. *coli* viability [37, 38]. It is possible that more of the 77 *N*. *meningitidis* proline stretch-containing proteins are essential for *N*. *meningitidis* viability, but there is currently no information available about essential *N*. *meningitidis* genes. Recently, it was reported that the stalling at the PPP and PPG motifs can be attenuated by the preceding amino acid residue [8, 9]. Other strong proline pausing stretches, including 16 DPP, 1 PPW, 22 PPD, 70 APP, and 25 PPN [6], are present in 113 proteins encoded in the *N*. *meningitidis* MC58 genome (S3 Table). The DPP, PPW, PPD, APP, and PPN motifs are included in 19 proteins with *E*. *coli* homologues that are essential [37, 38], and these proteins may also be involved in the viability of *N*. *meningitidis* cells. Other proline stretch-containing proteins may also be essential for *N*. *meningitidis* viability, thus causing the requirement for the EF-P(*Nm*) function, although their homologues are not essential in *E*. *coli*. Another possible reason for the requirement of the EF-P(*Nm*) function in *N*. *meningitidis* cells is that the synthesis of proline stretch-containing proteins in the absence of EF-P may be much less efficient than that in *E*. *coli* cells.

### *N*. *meningitidis* EarP is important for cell viability

**Fig 7:**
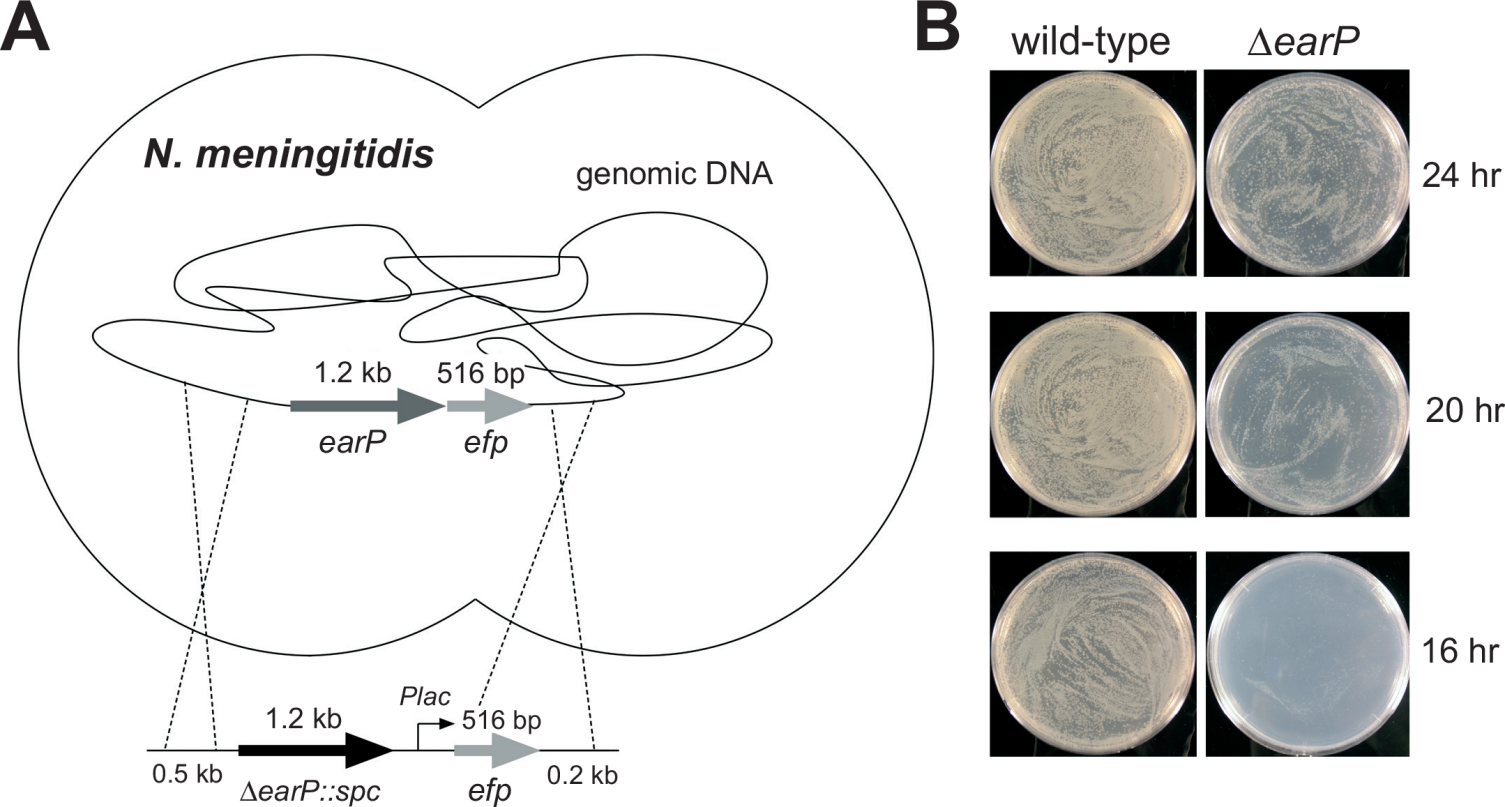
The *earP* gene is important, but not essential, for *N*. *meningitidis* viability. (A) Strategy for the *earP* deletion in *N*. *meningitidis*. (B) Growth of *N*. *meningitidis* HT1125 wild-type (left) and the *earP*-deleted cells (right).

In the same manner as for the *efp* gene, we attempted to disrupt the *earP* gene, encoding EarP, in the *N*. *meningitidis* genome (Fig 7A). *N*. *meningitidis* HT1125 cells were transformed with a fragment containing the *earP*-flanking regions and the spectinomycin resistance gene (*spc*), in order to disrupt the *earP* gene in the *N meningitidis* chromosome, and we obtained many spectinomycin-resistant (Spc^r^) colonies of strain HT1907, with the *ΔearP*::*spc* allele (Fig 7B, right). Nevertheless, the growth of the *N*. *meningitidis ΔearP* cells was much slower than that of the *N*. *meningitidis* HT1125 cells (Fig 7B, left). As described above, no colonies of the *efp* null mutant were obtained in the presence of the plasmid harboring the *efp*(R32K) or *efp*(R32A) mutant gene (Table 1). Taken together, we confirmed that the Arg32 residue in EF-P is essential, but the post-translational rhamnosyl modification of EF-P(Arg32) is not essential, for the viability of *N*. *meningitidis* cells. Therefore, EF-P(*Nm*) with the unmodified Arg32 can function to some extent on the *N*. *meningitidis* ribosome, although the activity is much lower than that of the rhamnosyl**–**EF-P(*Nm*). In contrast, the unmodified EF-P(*Nm*) did not function on the *E*. *coli* ribosome (Fig 6B and 6C and S7B and S7C Fig). Furthermore, as described above, the plasmid pMW-*Nm*E, containing the *N*. *meningitidis efp* gene, slowed the cell growth (S6 Fig), suggesting that the unmodified EF-P(*Nm*) is not only non-functional but also inhibitory for the *E*. *coli* ribosome. Consequently, the *N*. *meningitidis* ribosome is different from the *E*. *coli* ribosome, in that the unmodified EF-P(*Nm*) is not inhibitory but minimally functional. It is therefore important to clarify the difference in the precise mechanisms of proline stretch translation between the *E*. *coli* ribosome and the *N*. *meningitidis/S*. *oneidensis* ribosomes.

### The divergent post-translational modifications of EF-P may be due to evolutionary convergence

**Fig 8:**
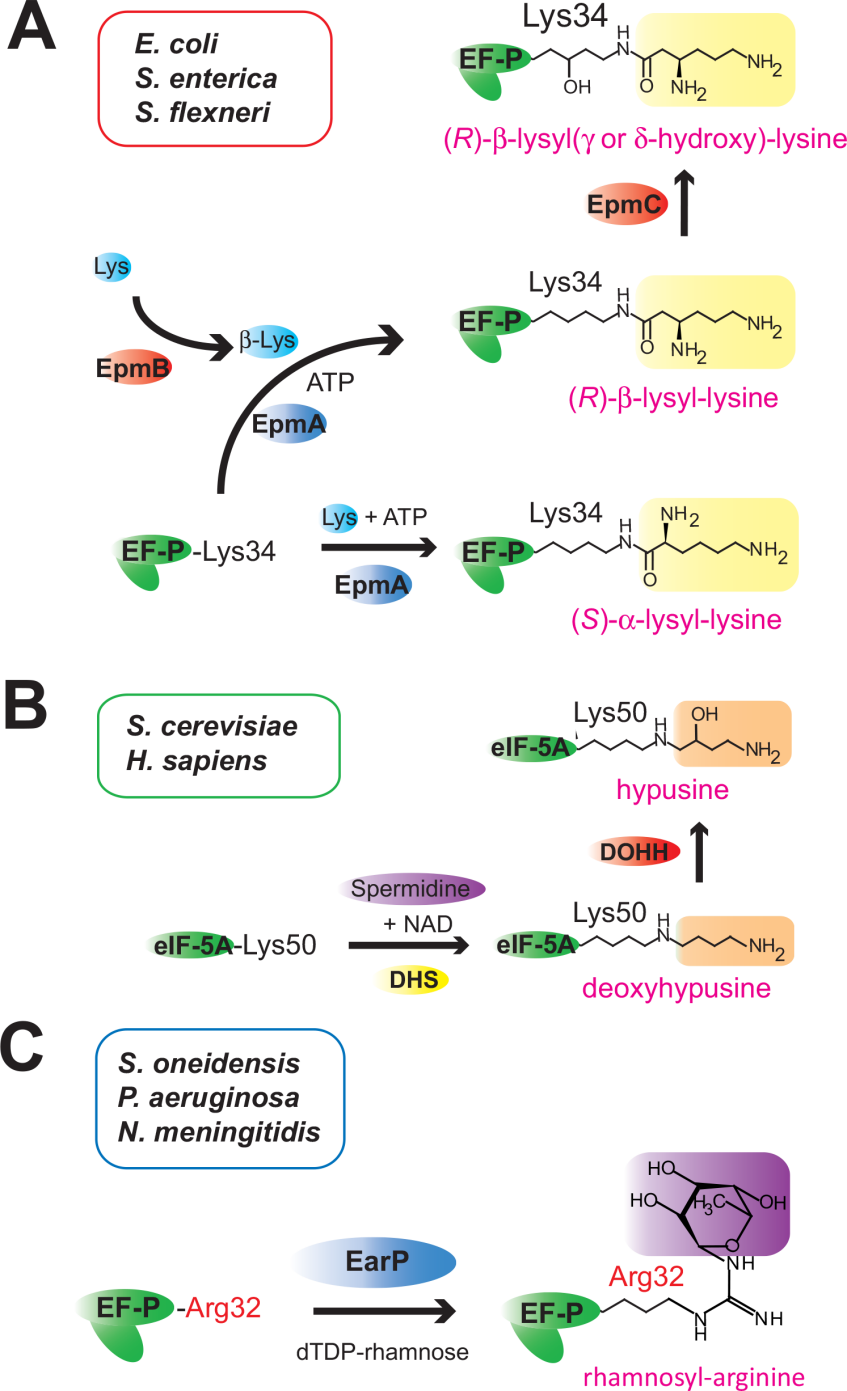
Post-translational modifications of EF-P and eIF5A. (A) β-Lysylation and subsequent hydroxylation of EF-P(Lys34) in *E*. *coli*, *S*. *enterica*, and *S*. *flexneri*. EpmB catalyzes the conversion of (*S*)-lysine (L-lysine) to (*R*)-β-lysine, and EpmA mainly uses (*R*)-β-lysine as the substrate for the production of (*R*)-β-lysyl–EF-P. β-Lys represents (*R*)-β-lysine. (B) Deoxyhypusine modification and subsequent hydroxylation of eIF5A(Lys50) in *H*. *sapiens* and *S*. *cerevisiae*. (C) Rhamnosyl modification of EF-P(Arg32) in *S*. *oneidensis*, *P*. *aeruginosa*, and *N*. *meningitidis*. The DUF2331 superfamily proteins (EarP) that catalyze the rhamnosyl modification of EF-P(Arg32) are encoded in the genomes of *N*. *meningitidis*, *N*. *gonorrhoeae* (accession code: AAW89632), *B*. *pertussis* (accession code: CAE42291), *B*. *cepacia* (accession code: AIO49016), *K*. *kingae* (accession code: EIC14610), *R*. *solanacearum* (accession code: CBJ51687), *P*. *aeruginosa* (accession code: WP_034078567), *P*. *fluorescens* (accession code: AIG05734), and *A*. *hydrophila* (accession code: ABK36714), but are not encoded in those of *B*. *burgdorferi*, *T*. *thermophilus*, or *D*. *radiodurans*.

Although the β-lysyl and rhamnosyl modification pathways are phylogenetically unrelated to each other, both post-translational modifications are analogous, in that the modified Lys34/Arg32 residue might thus be extended to reach the ribosomal peptidyltransferase center [35]. We propose that the long side chain at the Lys34/Arg32 position of EF-P is required for EF-P to rescue stalled ribosomes efficiently. Here, we suggest that such diverse post-translational modifications, including the β-lysylation/rhamnosylation of EF-P (and the hypusination of eIF5A) (Fig 8), are typical examples of “convergent evolution”. Evolutionary convergence creates analogous structures that have similar forms or functions, which were not present in the common ancestor. Previously, we proposed that the molecular mimicry between the lysyl modification of EF-P(*Ec*)(Lys34) by EpmA and the aminoacylation of tRNA(A76) by aaRS resulted from convergent evolution [18]. Likewise, such evolutionary convergence might have occurred in the post-translational EF-P/eIF5A modifications during evolution.

### The post-translational rhamnosyl modification of EF-P(Arg32) in *N*. *meningitidis* is a new antibiotic target

*N*. *meningitidis* is a Gram-negative diplococcus pathogen that colonizes the nasopharynx. It can spread into the bloodstream, where it causes septicaemia and furthermore induces meningitis when it reaches the cerebrospinal fluid. Although some of the factors involved in its pathogenesis have been identified (reviewed in [44]), many problems still remain to be resolved: *e*.*g*., the development of an appropriate antibiotic therapy for systemic meningococcal disease is urgently required. The present *in vivo* analyses using the EF-P-deficient *N*. *meningitidis* mutant revealed that EF-P(*Nm*) is important for *N*. *meningitidis* survival. Therefore, EF-P(*Nm*) may be a target for antibiotic drug development. In general, the three-dimensional structures of EF-Ps are mostly composed of convex surfaces, and it is therefore difficult to design chemical compounds that specifically bind to EF-P and inhibit it efficiently. However, the present study revealed that the post-translational modification of EF-P(*Nm*) is important, but not essential, for the growth of *N*. *meningitidis*, which is quite unusual among bacteria. Consequently, since the enzymatic catalytic site may have one or more pockets for substrate binding, and may generally be suitable for drug design and development (*i*.*e*., druggable), the rhamnosylation enzyme EarP(*Nm*) is an attractive target for antibiotic drug development toward the treatment of meningitis.

### The post-translational rhamnosyl modification of EF-P(Arg32) in other pathogenic bacteria

The deficiency in EF-P and/or its post-translational modification reportedly attenuate the virulence and infectivity of pathogenic bacteria [17, 24, 26, 35, 45, 46]. Besides *N*. *meningitidis*, the genomes of certain pathogenic β-proteobacteria and γ-proteobacteria encode the conserved EF-P(Arg32) and the rhamnosyl modification enzyme EarP (S4 Table). Therefore, the post-translational rhamnosyl modification might be equally important in other clinically relevant species, such as *Neisseria gonorrhoeae*, *Bordetella pertussis*, *Burkholderia pseudomallei*, and *Burkholderia cepacia*, because all of the EF-P proteins from these bacteria contain Arg32. As a limited number of pathogenic bacteria have the EF-P bearing Arg32 and the corresponding rhamnosylation enzyme homologue, the EF-P rhamnosylation pathway should be a target for new species-specific antibacterial agents against these pathogenic bacteria.

## Materials and Methods

Biochemical and molecular biological procedures were performed using commercially available materials, enzymes, and chemicals. The polyclonal antibody against EF-P(*Ec*) was purchased from Keari Bio (Osaka, Japan).

### Bacterial strains

*N*. *meningitidis* strains HT1125 and its derivatives, and H44/76 and its derivatives were stored at −80°C, and routinely grown on GC agar plates at 37°C in 5% CO_2_ [47, 48]. *E*. *coli* strains BL21-Gold(DE3), BW25113, and JW4107 (BW25113 Δ*efp*) were grown on plates or liquid culture of LB broth, Miller (Nacalai Tesque) at 37°C.

### Purification of the endogenous EF-P from *N*. *meningitidis* cells

All steps were performed at 4°C. Frozen *N*. *meningitidis* HT1125 cells (13 g) were suspended in B-PER bacterial protein extraction reagent (Takara), and disrupted by freeze-thawing. The crude cell extract (50 ml, 489 mg total protein) was centrifuged at 10,000 × *g* for 20 min. After the resulting supernatant was dialyzed overnight against 50 mM potassium phosphate buffer (pH 7.4), containing 1 mM DTT and 0.1 mM PMSF (buffer A), the solution was applied to a column of DEAE-Sephacel (50 ml, GE Healthcare) equilibrated with buffer A. The column was washed and then developed with a 0 to 0.4 M NaCl gradient. Active fractions of the eluate were identified with a polyclonal antibody against EF-P(*Ec*), bearing cross-reactivity against EF-P(*Nm*) (Fig 2B), collected, and dialyzed against buffer A. The DEAE-Sephacel fraction (30.3 mg total protein) was applied to a HiTrap Q HP column (GE Healthcare) equilibrated with buffer A. The column was washed, and the proteins were eluted by a linear gradient of 0 to 0.5 M NaCl. Active fractions of the eluate were identified with the anti-EF-P(*Ec*) polyclonal antibody, collected, and dialyzed against buffer A. To the HiTrap Q fraction (3.49 mg total protein), (NH)_2_SO_4_ was added to a final concentration of 1 M. This solution was loaded on a HiTrap Butyl HP column (GE Healthcare), equilibrated with buffer A containing 1 M (NH)_2_SO_4_. After the column was washed with buffer A containing 1 M (NH)_2_SO_4_, the proteins were eluted with a linear gradient of 1 to 0 M (NH)_2_SO_4_. The eluted EF-P(*Nm*) proteins were collected, dialyzed against 20 mM potassium phosphate buffer (pH 7.4) containing 0.15 M NaCl and 10 mM β-mercaptoethanol, flash cooled with liquid nitrogen, and stored at –80°C (1.06 mg total protein) until use.

### Cloning, overexpression, and purification of recombinant *N*. *meningitidis* EF-P

The *efp* gene (NMH_0798, accession code: EFV64285) encoding EF-P(*Nm*) was PCR-amplified from *N*. *meningitidis* H44/76 genomic DNA by ExTaq DNA polymerase (Takara), and cloned into the *Nde*I and *Bam*HI sites of the pET23 and pET28 vectors to construct the plasmids pET-*Nm*E1 and pET-*Nm*E2, respectively (S1 Table). *E*. *coli* BL21-Gold(DE3) cells were transformed with the plasmid pET-*Nm*E1, containing a non-tagged *N*. *meningitidis efp* gene. The cells harboring pET-*Nm*E1 were grown in LB broth (Miller) medium to an OD_600_ of 0.6, and then protein expression was induced with 1 mM IPTG at 37°C for 4 hr. The cells were harvested, sonicated, and centrifuged to remove the cell debris. The supernatant, containing the recombinant EF-P(*Nm*) protein, was dialyzed against 20 mM potassium phosphate buffer (pH 7.4), containing 150 mM NaCl and 10 mM β-mercaptoethanol, treated with thrombin (0.1 mg protein per unit) at 4°C overnight, and subjected to MALDI-TOF MS or ESI-MS analyses. The purified EF-P(*Nm*) protein was flash-cooled with liquid nitrogen, and stored at −80°C. The gene encoding the EarP(*Nm*) homologue (accession code: LC059993) was PCR-amplified from *N*. *meningitidis* HT1125 genomic DNA by ExTaq DNA polymerase (Takara), and cloned into the *Bam*HI and *Hind*III sites of pET-*Nm*EFP2, and the *Nde*I and *Hind*III sites of pET28, to construct the plasmids pET-*Nm*ED and pET-*Nm*D, respectively (S1 Table). Expression, purification, and MS analyses of the EarP(*Nm*)-modified recombinant EF-P(*Nm*) were performed as described below.

### Post-translational modification assays

*E*. *coli* BL21-Gold(DE3) cells were transformed with the plasmid pET-*Nm*E2, containing the *efp* gene encoding EF-P with an N-terminal hexahistidine tag (His_6_, MGSSHHHHHHSSGLVPRGSH), and the recombinant EF-P(*Nm*) protein was overexpressed as described above. The crude extract (100 μl, 1.5 mg/ml protein) was prepared from the recombinant EF-P(*Nm*)-producing cells, mixed with the crude cell extract (40 μl, 4.78 mg/ml protein) from *N*. *meningitidis* HT1125 cells, and incubated at 37°C overnight. The His_6_-tagged EF-P(*Nm*) was purified by batch chromatography on Ni-Sepharose (GE Healthcare), as follows. Ni-Sepharose (25 μl) was added to this solution, which was incubated on a rotary shaker at 4°C for 2 hr. The gel was washed three times with 50 mM potassium phosphate buffer (pH 7.4), containing 150 mM NaCl and 10 mM β-mercaptoethanol (buffer C), and EF-P(*Nm*) was eluted with buffer C containing 350 mM imidazole. The purified EF-P(*Nm*) was dialyzed against 20 mM potassium phosphate buffer (pH 7.4), containing 150 mM NaCl and 10 mM β-mercaptoethanol, treated with thrombin (0.1 mg protein per unit) at 4°C overnight, and analyzed by MALDI-TOF MS.

### Western blot analysis

The crude extract prepared from *N*. *meningitidis* cells and the fractions from each column chromatography step were resolved by 10–20 % SDS-PAGE, and transferred to an Immobilon P membrane (Millipore). EF-P(*Nm*) was recognized using a polyclonal antibody against EF-P(*Ec*) and a horseradish peroxidase (HRP)-conjugated anti-rabbit IgG antibody from donkey (GE Healthcare). Western blot analyses were performed as described previously [49, 50], with some modifications.

### Mass spectrometry analyses

The molecular masses of the *N*. *meningitidis* endogenous and recombinant EF-P proteins were determined with the aid of a Voyager-DE STR MALDI-TOF mass spectrometer (Applied Biosystems) and a TOF/TOF5800 system (AB SCIEX). The endogenous and recombinant EF-P(*Nm*) proteins were subjected to SDS-PAGE, followed by staining with Coomassie Brilliant Blue or SimplyBlue SafeStain (Life Technologies). The protein bands (about 30 kDa) were excised and digested in the gel with endoproteinase AspN (Sequencing Grade, Basel, Switzerland). The digests were separated on a column of Inertsil ODS-3 (1 × 100mm; GL Sciences Inc., Tokyo) with a model 1100 series liquid chromatography system (Agilent Technologies, Waldbronn, Germany), using solvents A and B, which were 0.085% (v/v) aqueous trifluoroacetic acid (TFA) and 0.075% (v/v) TFA in 80% (v/v) acetonitrile, respectively. The peptides were eluted at a flow rate of 20 μl/min, using a linear gradient of 0–60% solvent B. The two peptides selected from the peptide map of these digests were subjected to Edman degradation, using a Procise cLC protein sequencing system (Applied Biosystems). The peptide from the endogenous EF-P(*Nm*), with the sequence DPMVVQKTEYIkggXssaxv (X designates a modified arginine residue), was further digested with trypsin or Achromobacter protease I (API; a gift from Dr. Masaki, Ibaraki University [51]). The digest was subjected to MALDI-TOF MS on an Ultraflex mass spectrometer (Bruker Daltonics, Bremen, Germany) in the reflector mode, using 2, 5-dihydroxybenzoic acid (DHB) as the matrix. The API digest was further purified on a porous graphitic carbon column (Hypercarb, 1.0 × 100 mm, 3 μm, Thermo Fisher Scientific Inc., San Jose, CA) with a model 1100 series liquid chromatography system (Agilent Technologies) at a flow rate of 20 μl/min, using a linear gradient of 1–50% solvent B. The purified peptide was subjected to MALDI-TOF MS and MS/MS analyses, using an Ultraflex system.

### Sugar components and amino acid analysis

The Hypercarb column purified peptide was subjected to a sugar components analysis and an amino acid analysis. The sugar components analysis was performed after hydrolysis with 4 M TFA for 3 hr at 100°C. The sample was *N*-acetylated, labeled with a fluorescent label (ABEE, 4-aminobenzoic acid ethyl ester), and then analyzed using a Honenpak C18 column (4.6 mm i.d. × 75 mm) from J-Oil Mills Inc., developed with 0.2 M potassium borate buffer (pH 8.9) containing 7% acetonitrile, along with a standard kit from J-Oil Mills Inc. containing 11 monosaccharides [52]. For amino acid analysis, the peptide was hydrolyzed in the vapor gas phase in constantly boiling conc. HCl at 110°C for 24 hr. The hydrolysate was analyzed by the pre-label method, using 6-aminoquinolyl-N-hydroxysuccinimidyl carbamate for fluorescence detection, ion-pair chromatography with a reversed-phase column, and an ultra-high-pressure high-performance liquid chromatography (UHPLC) system [53].

### Complementation analysis of the *efp* null mutant of *N*. *meningitidis*

A 1.1-kb DNA fragment containing the *efp* gene (0.5 kb) and its upstream (0.3 kb) and downstream (0.3 kb) regions was amplified with the primers efp-5 (5′-AACAACATCAAAACGGCTGGCGGCA-3′) and efp-6 (5′-CGGAGAAATGCTTGAAAACCAATC-3′) by PrimeStar Max DNA polymerase (Takara Bio, Japan) using *N*. *meningitidis* HT1125 genomic DNA as the template, and cloned into the *Sma*I site of pMW119 (Nippon Gene) to construct the plasmid pMW-*Nm*E (5.3 kb). The 1.1-kb *Kpn*I-*Bam*HI DNA fragment of pMW-*Nm*E was subcloned into the same sites of pHT261 [54] (S1 Table), an IncQ plasmid, to construct pHT969. The plasmid pHT969 was further mutagenized with PrimeStar Max DNA polymerase to obtain the plasmids pHT971, harboring *N*. *meningitidis efp*(R32K), and pHT972, harboring *N*. *meningitidis efp*(R32A). The introduction of pHT969, pHT971, and pHT972 into *N*. *meningitidis* HT1125 was performed as described previously [47].

To construct the *N*. *meningitidis* mutant with the deletion of the *efp* gene, a 1.6-kb DNA fragment containing the *efp* gene (0.5 kb) and its upstream (0.6 kb) and downstream (0.5 kb) regions was amplified with the primers efp-1 (5′- TGAAAGGCTGCATCTGATGCCTTCGCCGCA-3′) and efp-2 (5′- CGGACTGTCTGTTTGCCCTTTCCCATCACG-3′) by PrimeStar Max DNA polymerase, using *N*. *meningitidis* HT1125 genomic DNA as the template. The fragment was cloned into the *Sma*I site of pMW119 (Nippon Gene), to construct the plasmid pMW-*Nm*E2 (5.8 kb). The 5.3 kb DNA fragment from pMW-*Nm*E2, which does not contain the *efp* coding region, was amplified again by PrimeSTAR Max DNA polymerase (Takara), and ligated to an erythromycin resistance gene (*ermC*) to obtain pMW-*Nm*E2-Erm. The linearized DNA fragment (2.1 kb) containing the *ermC* gene and the *efp* flanking regions (*Δefp*::*ermC*) was amplified from pMW-*Nm*E2-Erm, and transformed into HT1125 in the presence or absence of plasmids harboring the wild-type *N*. *meningitidis efp*, *efp*(R32K), and *efp*(R32A) genes. Transformation was performed as described previously, with some modifications: 1 μl (50 ng) of the PCR product from pMW-*Nm*E2-Erm was added to 1 ml *N*. *meningitidis* suspension in GC broth [47], corresponding to 0.2 OD_600_, and rotated at 37°C for 3 hr. A 100 μl portion of the suspension was spread onto GC agar medium [47], containing 5 μg/ml chloramphenicol and 4 μg/ml erythromycin, and incubated overnight at 37°C in 5% CO_2_. The number of erythromycin-resistant (Erm^r^) colonies was counted. At the same time, to examine the *efp* allele, 16 Erm^r^ colonies were selected and colony PCR was performed with the primers efp-1 and efp-2, which only amplify the chromosomal *efp* allele, using Tks Gflex DNA polymerase (Takara Bio). The amplified DNA was fractionated on a 0.7% agarose gel. The lengths of the wild-type *efp* and *Δefp*::*ermC* alleles were 2 kb and 2.5 kb, respectively. In this study, Erm^r^ colonies appeared in all of the *N*. *meningitidis* transformation experiments, probably because the *Δefp*::*ermC* gene was inserted into unidentified allele(s) other than *efp* due to unexpected recombination. Therefore, the number of *Δefp*::*ermC* alleles (*n*) in 16 Erm^r^ colonies was counted, and the ratio *n*/16 was multiplied by the total number of Erm^r^ colonies, in order to estimate the total number of *Δefp*::*ermC* mutants.

### Examination of the essentiality of the inducible *efp* in *N*. *meningitidis*

The *N*. *meningitidis* strain with an inducible *efp* gene was constructed as follows. The 1.1-kb *Bam*HI-*Kpn*I DNA fragment of pMW-*Nm*E was subcloned into the same sites of pTTQ19 [55], to construct the plasmid pHT1095. To reduce the expression level, the translational start codon (ATG) in the *efp* gene was replaced with TTG by site-directed mutagenesis, to construct the plasmid pTTQ-*Ptac*-Δ200-TTG-*Nmefp*. The pTTQ-*Ptac*-Δ200-TTG-*Nmefp* was digested with *Sca*I and *Alw*NI, and the *Alw*NI-*Sca*I DNA fragment was blunt-ended and cloned into the blunted *Sca*I and *Pst*I sites of the IncQ plasmid pGSS33 [47], to construct pHT1139. The pHT1139 plasmid was introduced into *N*. *meningitidis* H44/76 cells, to construct the transformant H44/76/pHT1139.

The endogenous *efp* gene in the *N*. *meningitidis* genome was inactivated by the introduction of the *Δefp*::*ermC* mutation, as follows. The linearized DNA fragment (2.1 kb), containing the *ermC* gene and the *efp* flanking regions (*Δefp*::*ermC*), was amplified from pMW-*Nm*E2-Erm, and transformed into the *N*. *meningitidis* H44/76/pHT1139 cells. Transformation was performed as described previously, with some modifications: 1 μl (50 ng) of the PCR product amplified from pMW-*Nm*E2-Erm was added to a 1 ml suspension of the *N*. *meningitidis* H44/76/pHT1139 cells in GC broth [47], corresponding to 0.2 OD_600_, rotated at 37°C for 8 hr, spread onto GC agar medium [47], containing 5 μg/ml chloramphenicol and 4 μg/ml erythromycin, and incubated overnight at 37°C in 5% CO_2_, to obtain *N*. *meningitidis* HT1913/pHT1139. To examine the *efp* allele in the HT1913 genome, 16 Erm^r^ colonies were selected and colony PCR was performed with the primers efp-1 and efp-2, which amplify the chromosomal *efp* allele only, using Tks Gflex DNA polymerase (Takara Bio). The amplified DNA was fractionated on a 0.7% agarose gel. The lengths of the wild-type *efp* and *Δefp*::*ermC* alleles were 2 kb and 2.5 kb, respectively.

The genomic *N*. *meningitidis efp* gene was also inactivated by the introduction of the *efp(R32opal)* mutation, as follows. pMW-NmE2 was mutagenized with PrimeStar Max DNA polymerase to obtain the plasmid pHT1094, harboring *N*. *meningitidis efp*(*R32opal*) (6.3kb). The 5.3-kb DNA fragment was amplified 150 bp downstream of the stop codon of the *efp* gene, with the primers efp-25 (TTTGTCGGGATTGCGTTCACGGTT) and efp-26 (CGTTTTTAGACATCCATTTTGACGAAA) by PrimeStar Max DNA polymerase, and ligated to the 0.8 kb DNA fragment of the erythromycin resistance gene (*ermC*), to construct the plasmid pHT1098. A 2.3-kb DNA fragment, containing the *efp(R32opal)* gene (0.5 kb), and the *ermC* gene (0.8 kb) and its upstream (0.8 kb) and downstream (0.6 kb) regions, was transformed into *N*. *meningitidis* H44/76/pHT1139 cells. The resultant Erm^r^ colonies were selected and named *N*. *meningitidis* HT1914/pHT1139, and the *efp(R32opal)* mutation was confirmed by direct PCR sequencing. The essentiality of the *N*. *meningitidis efp* gene was examined as follows. *N*. *meningitidis* HT1913/pHT1139 and HT1914/pHT1139 were grown on GC agar, containing 0.5 mM IPTG, at 37°C in a 5% CO_2_ atmosphere overnight. The colonies were collected and suspended in 1 ml PBS, at an OD_600_ of 0.01. The bacteria were harvested by centrifugation and resuspended in 1 ml PBS. This procedure was repeated five times. A 100 μl portion of the bacterial suspension was plated onto GC agar with or without 0.5 mM IPTG, and the plates were observed after an incubation at 37°C in a 5% CO_2_ atmosphere for 20 hrs.

### Analysis of the *earP* null mutant of *N*. *meningitidis*

A 2.5-kb DNA fragment, containing the *earP* (1.2 kb) and *efp* (0.5 kb) genes and their upstream (0.5 kb) and downstream (0.2 kb) regions, was amplified with the primers miaA-6 (5′-GGCGGTCGGCCCGAGCAGGGCAA-3′) and efp-down-2 (5′- CGGAGAAATGCTTGAAAACCAATC-3′) by PrimeStar Max DNA polymerase (Takara Bio, Japan), using *N*. *meningitidis* HT1125 genomic DNA as the template, and cloned into the *Sma*I site of pMW119 (Nippon Gene), to construct the plasmid pHT1088 (6.7 kb). Since the ATG start codon of the *efp* gene is located only 43 bp downstream of the stop codon of *earP* gene, we speculated that the *earP* and *efp* genes could be co-transcribed. To avoid the polar effect, the *earP* null mutant was constructed as follows. The 4.5-kb DNA fragment of pHT1088, which lacks the region encoding the *earP* and *efp* genes, was amplified by PrimeStar Max DNA polymerase. In addition, a 1-kb DNA fragment of pMW-*Nm*E in which the *efp* gene was fused to the *E*. *coli lac* promoter, and a 1-kb DNA fragment of the spectinomycin resistance gene (*spc*) were also amplified, using PrimeStar Max DNA polymerase. These three fragments were fused by In Fusion cloning (Clontech) to construct pHT1089, containing the *ΔearP*::*spc-Plac-efp* genes. To construct the *N*. *meningitidis ΔearP*::*spc-Plac-efp* mutant strain HT1907, the linearized DNA fragment (2.7 kb), containing the *ΔearP*::*spc-Plac-efp* genes, was amplified from pHT1089, and transformed into *N*. *meningitidis* HT1125 cells. Transformation was performed as described previously [47]. Spc^r^ colonies were selected, and the *ΔearP*::*spc-Plac-efp* allele in the *N*. *meningitidis* genome was confirmed by PCR.

### Expression of a proline stretch-containing protein in EF-P-deficient *E*. *coli* cells

The *E*. *coli* Flk and GntX expression plasmids (pCA24N-derived ASKA library plasmid) were obtained from the National Bio-Resource Project (NBRP), Japan [55]. The DNA fragment encoding EarP(*Nm*) was PCR-amplified from *N*. *meningitidis* HT1125 genomic DNA by PrimeStar Max DNA polymerase (Takara), and cloned into pMW-*Nm*E to construct the plasmid pMW-*Nm*ED (S1 Table). The DNA fragment encoding *E*. *coli* EF-P, EpmA, and EpmB was PCR-amplified from the plasmid pACTK-EGY [18] by PrimeStar Max DNA polymerase (Takara), and cloned into pMW119 to construct the plasmid pMW-*Ec*EGY. The plasmids pMW119, pMW-*Nm*E, pMW-*Nm*ED, and pMW-*Ec*EGY were co-transformed with the Flk plasmid into *E*. *coli* BW25113 and Δ*efp* cells. The cells harboring these plasmids were grown in LB broth (Miller) medium to an OD_600_ of 0.6, and then protein expression was induced with 1 mM IPTG at 37°C for 6 hr. The cells were harvested, and the expressed Flk protein with the His_6_-tag (MRGSHHHHHHTDPALRP) and the EF-P protein were analyzed by SDS-PAGE and western blotting, using an anti-His_6_ antibody and a polyclonal antibody against EF-P(*Ec*).

### Data deposition

The nucleotide sequences of EF-P and EarP from *N*. *meningitidis* strain HT1125 have been deposited in the DNA Data Bank of Japan (DDBJ) (accession codes: LC059994 and LC059993, respectively).

## Supporting Information

S1 Fig Purification of *N*. *meningitidis* endogenous EF-P by hydrophobic interaction chromatography. (A) Elution profile of the endogenous EF-P(*Nm*), fractionated by HiTrap Butyl column chromatography. The absorbances at 280 (A_280_) and 260 nm (A_260_), and the concentration of ammonium sulfate are shown by the red, blue, and green lines, respectively. The endogenous EF-P(*Nm*) was collected from the flow-through fractions. (B, C) The protein fractions were analyzed by SDS-PAGE (B) and western blotting using a polyclonal antibody against EF-P(*Ec*) (C). The numbers above each lane are the fraction numbers.
(PDF)

S2 Fig Structure-based sequence alignment of EF-P and a/eIF5A. The amino acid sequences were aligned using the program CLUSTAL W [56], and then partly optimized and adjusted manually. Highly conserved residues between EF-P and a/eIF5A are colored red. Highly conserved residues in EF-P are colored orange, and those in a/eIF5A are colored sky blue. The rhamnosylated Arg32 of *N*. *meningitidis* EF-P is highlighted with a purple asterisk above the sequence alignment. Dashes represent breaks in the actual amino acid sequences of the respective proteins, to allow sequence alignment with *N*. *meningitidis* EF-P. *Nm*EFP, *N*. *meningitidis* EF-P (EFV64285); N. *Ec*EFP, *E*. *coli* EF-P (AAA97046); *Ng*EFP, *Neisseria gonorrhoeae* EF-P (YP_208043); *Bp*EFP, *Bordetella pertussis* EF-P (NP_880680); *Bc*EFP, *Burkholderia cepacia* EF-P (WP_014897640); *Bb*EFP, *Borrelia burgdorferi* EF-P (YP_005806412); *Tt*EFP, *Thermus thermophilus* EF-P (BAD70948); *Dr*EFP, *Deinococcus radiodurans* EF-P (AAF09709); *Hs*IF5, *Homo sapiens* eIF5A (AAH80196); *Sc*IF5, *Saccharomyces cerevisiae* eIF5A (AAA34425); *Mj*IF5, *Methancaldococcus jannaschii* aIF5A (C64453). An overview of a ribbon diagram of the crystal structure of *E*. *coli* EF-P (left) and a structural model of *N*. *meningitidis* EF-P (right) built by the Phyre2 server [57], using *P*. *aeruginosa* EF-P (PDB code: 3OYY) as the template, and visualized by the PyMOL viewer (https://www.pymol.org/). The Lys34 residue in EF-P(*Ec*) and the corresponding residue (Arg32) in EF-P(*Nm*) are shown as stick models. Secondary structure assignments (α-helices, 3_10_-helices, and β-sheets) in EF-P(*Ec*) are represented as α, η, and β, respectively. The α-helices, 3_10_-helices, and β-sheets are colored deep purple, dark olive, and green, respectively.
(PDF)

S3 Fig The activity to modify recombinant EF-P(*Nm*) exists in the crude extract of *N*. *meningitidis* cells. (A) A crude extract prepared from *E*. *coli* cells producing recombinant EF-P(*Nm*) was incubated with the *N*. *meningitidis* crude extract. The extract-treated recombinant EF-P(*Nm*) was purified, the His_6_-tag was cleaved with thrombin, and the protein was subjected to MALDI-TOF MS analysis. Lane 1, molecular mass standards; lane 2, crude extract of *E*. *coli* cells producing EF-P(*Nm*) (CE1); lane 3, crude extract of *N*. *meningitidis* (CE2); lane 4, molecular mass standards; lane 5, mixture of CE1 and CE2; lane 6, purified recombinant EF-P(*Nm*). (B, C) The molecular mass of the CE2-treated recombinant EF-P(*Nm*) (obsd: 21,301.17) is larger by 148.6 Da than that of the recombinant EF-P(*Nm*) without the incubation with CE2 (calcd: 21,161.02, obsd: 21,152.62). The 149 Da mass increase is comparable to the difference between the endogenous EF-P(*Nm*) (obsd: 21,034.74) and the recombinant EF-P(*Nm*) (obsd: 20,887.39). The peaks with masses around 17,000–18,000 Da are presumed to be degradation products.
(PDF)

S4 Fig Sequence alignment of EarP of *N*. *meningitidis* strains HT1125, H44/76, and MC58. EarP of *N*. *meningitidis* HT1125 has 14 different residues from those of *N*. *meningitidis* H44/76 and MC58.
(PDF)

S5 Fig MS analysis of the modified and unmodified EF-P(*Nm*). MALDI-TOF MS spectra of the recombinant EF-P(*Nm*) purified from *E*. *coli* cells producing only EF-P(*Nm*) (Fig 5A, lane 6) (A), and the recombinant EF-P(*Nm*) purified from *E*. *coli* cells producing both EF-P(*Nm*) and EarP(*Nm*) (Fig 5A, lane 7) (B).
(PDF)

S6 Fig Complementation analysis of *E*. *coli efp* null mutant with *N*. *meningitidis* EF-P. Plasmids pMW119, pMW-*Nm*E (a pMW119-derived plasmid for expressing EF-P(*Nm*)), pMW-*Nm*ED (a pMW119-derived plasmid for expressing EF-P(*Nm*) and EarP(*Nm*)), and pMW-*Ec*EGY (a pMW119-derived plasmid for expressing EF-P(*Ec*), EpmA, and EpmB) were introduced into the *E*. *coli* K12 strain BW25113 and the Δ*efp* strain JW4107 (BW25113 Δ*efp*::*kan*), and the growth of these strains was compared on LB-Amp plates at 37°C. (A) BW25113/pMW119 (B) BW25113/pMW-*Nm*E (C) BW25113/pMW-*Nm*ED (D) BW25113/pMW-*Ec*EGY (E) JW4107/pMW119 (F) JW4107/pMW-*Nm*E (G) JW4107/pMW-*Nm*ED (H) JW4107/pMW-*Ec*EGY. (I) The growth curves of these cells in LB broth (Miller) medium in the presence of Amp (50 μg/ml) at 37°C are also shown.
(PDF)

S7 Fig Restoration of production of the *E*. *coli* GntX protein, containing proline stretches, in *efp*-deficient cells. (A) Amino acid sequence of *E*. *coli* GntX. Proline stretches in GntX are colored red. (B) Plasmids pMW119, pMW-*Nm*E, pMW-*Nm*ED, and pMW-*Ec*EGY were cotransformed with the GntX plasmid into *E*. *coli* BW25113 and JW4107 (BW25113 Δ*efp*::*kan*) cells. Whole-cell proteins were subjected to SDS-PAGE, and analyzed for the production of the full-length GntX protein. Lane 1: molecular mass standards; lane 2: BW25113/pMW119; lane 3: BW25113/pMW-*Nm*E; lane 4: BW25113/pMW-*Nm*ED; lane 5: BW25113/pMW-*Ec*EGY; lane 6: JW4107/pMW119; lane 7: JW4107/pMW-*Nm*E; lane 8: JW4107/pMW-*Nm*ED; lane 9: JW4107/pMW-*Ec*EGY; lane 10, MagicMark molecular mass standards. (C) The GntX protein was recognized by an anti-His_6_-antibody. (D) Detection of the expressed *N*. *meningitidis* EF-P using an anti-EF-P antibody.
(PDF)

S1 Table Strains and plasmids. (DOCX)

S2 Table Sequences and calculated masses of *N*. *meningitidis* EF-P. The residues from the pET28-derived His_6_-tag are shown in blue letters. *N*. *meningitidis* HT1125 EF-P has a point mutation in domain III, where the Val173 residue in *N*. *meningitidis* H44/76 EF-P is replaced by Leu173, as shown in red letters. The amino acid sequence identities of HT1125 EF-P with those of H44/76 and MC58 are 99.5%. The Arg32 residues are highlighted in purple.
(DOCX)

S3 Table Proline stretch-containing proteins encoded in the *N*. *meningitidis* MC58 genome. All of the proteins encoded in the *N*. *meningitidis* MC58 genome were searched for the presence of proline stretches. Proline–proline–proline–proline (PPPP), proline–proline–proline (PPP), aspartic acid–proline–proline (DPP), proline–proline–tryptophan (PPW), proline–proline–aspartic acid (PPD), alanine–proline–proline (APP), proline–proline–asparagine (PPN), and proline–proline–glycine (PPG) residues are colored red.
(DOCX)

S4 Table EF-P modification enzymes in pathogenic bacteria. Pathogenic bacteria containing EF-P(Arg32) and rhamnosyl modification enzymes (EarP homologues) are colored red, and those containing EF-P(Lys32/33/34), EpmA, and EpmB are colored blue. *B*. *burgdorferi* containing EF-P(Arg32) but lacking a EarP homologue is colored orange.
(DOCX)

## Abbreviations

aaRS: aminoacyl-tRNA synthetase
API: Achromobacter protease I
CHCA: α-cyano-4-hydroxycinnamic acid
DHB: 2,5-dihydroxybenzoic acid
ESI-Q-TOF MS: electrospray ionization quadrupole time-of-flight mass spectrometry
MALDI-TOF MS: matrix assisted laser desorption ionization time-of-flight mass spectrometry
MS/MS: tandem mass spectrometry
PMF: peptide mass fingerprinting
TFA: trifluoroacetic acid
UHPLC: ultra-high-pressure high-performance liquid chromatography
