# Supplementary material for: Neisseria meningitidis Translation Elongation Factor P and Its Active-Site Arginine Residue Are Essential for Cell Viability
Source: PLoS One. 2016 Feb 3;11(2):e0147907. doi: 10.1371/journal.pone.0147907 (PMC4739656; doi:10.1371/journal.pone.0147907)
Supplement: S1 Fig — (A) Elution profile of the endogenous EF-P(Nm), fractionated by HiTrap Butyl column chromatography. The absorbances at 280 (A280) and 260 nm (A260), and the concentration of ammonium sulfate are shown by the red, blue, and green lines, respectively. The endogenous EF-P(Nm) was collected from the flow-through fractions. (B, C) The protein fractions were analyzed by SDS-PAGE (B) and western blotting using a polyclonal antibody against EF-P(Ec) (C). The numbers above each lane are the fraction numbers. (PDF) [file pone.0147907.s001.pdf]

Yanagisawa *et al.*, Fig. S1

A

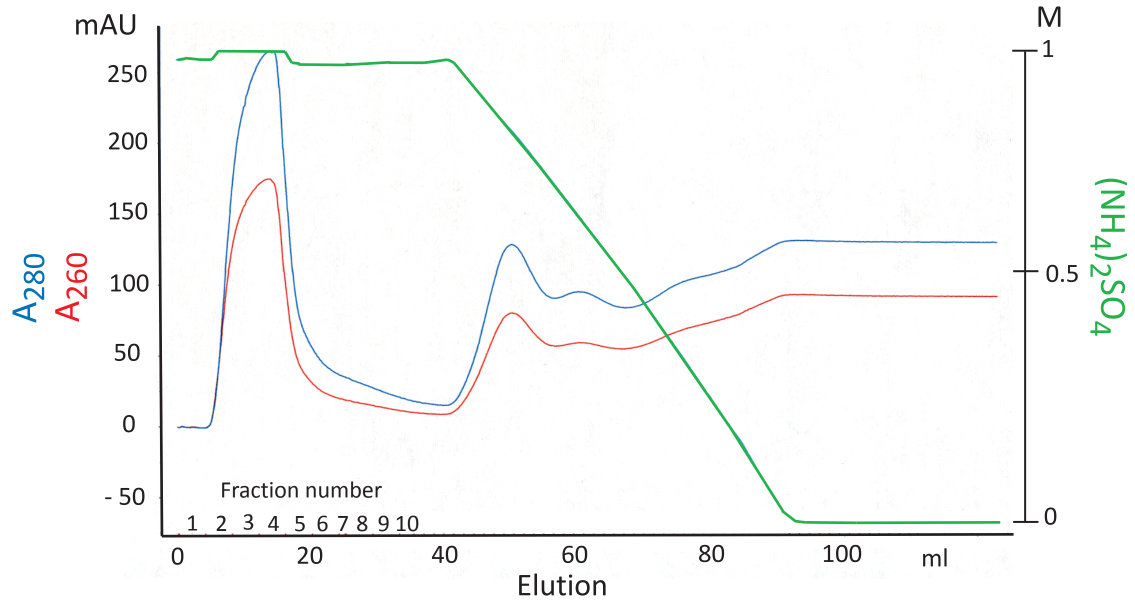

B

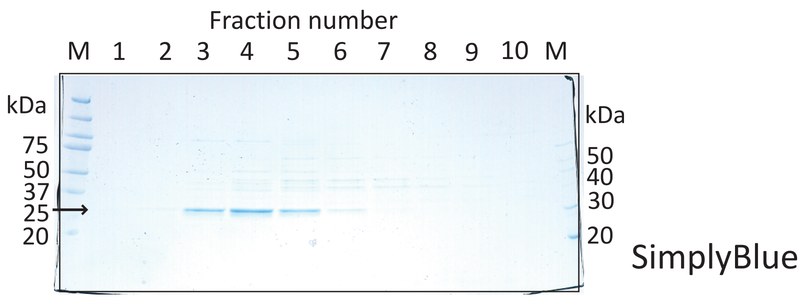

C

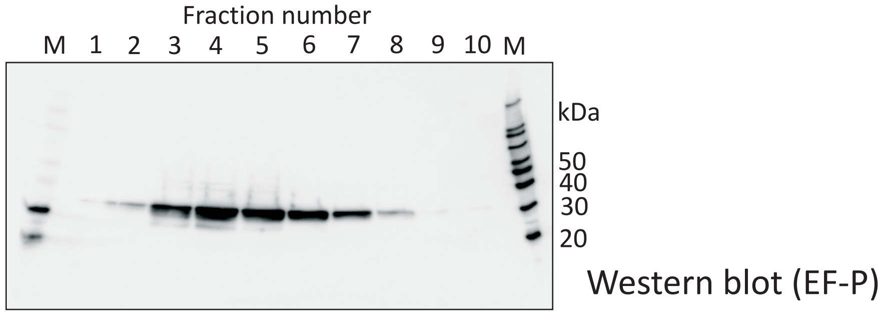

Fig. S1. Purification of *N. meningitidis* endogenous EF-P by hydrophobic interaction chromatography.

(A) Elution profile of the endogenous EF-P(*Nm*), fractionated by HiTrap Butyl column chromatography. The absorbances at 280 (A_{280}) and 260 nm (A_{260}), and the concentration of ammonium sulfate are shown by the red, blue, and green lines, respectively. The endogenous EF-P(*Nm*) was collected from the flow-through fractions. (B, C) The protein fractions were analyzed by SDS-PAGE (B) and western blotting using a polyclonal antibody against EF-P(*Ec*) (C). The numbers above each lane are the fraction numbers.
