# Supplementary material for: Neisseria meningitidis Translation Elongation Factor P and Its Active-Site Arginine Residue Are Essential for Cell Viability
Source: PLoS One. 2016 Feb 3;11(2):e0147907. doi: 10.1371/journal.pone.0147907 (PMC4739656; doi:10.1371/journal.pone.0147907)
Supplement: S2 Fig — The amino acid sequences were aligned using the program CLUSTAL W [56], and then partly optimized and adjusted manually. Highly conserved residues between EF-P and a/eIF5A are colored red. Highly conserved residues in EF-P are colored orange, and those in a/eIF5A are colored sky blue. The rhamnosylated Arg32 of N. meningitidis EF-P is highlighted with a purple asterisk above the sequence alignment. Dashes represent breaks in the actual amino acid sequences of the respective proteins, to allow sequence alignment with N. meningitidis EF-P. NmEFP, N. meningitidis EF-P (EFV64285); N. EcEFP, E. coli EF-P (AAA97046); NgEFP, Neisseria gonorrhoeae EF-P (YP_208043); BpEFP, Bordetella pertussis EF-P (NP_880680); BcEFP, Burkholderia cepacia EF-P (WP_014897640); BbEFP, Borrelia burgdorferi EF-P (YP_005806412); TtEFP, Thermus thermophilus EF-P (BAD70948); DrEFP, Deinococcus radiodurans EF-P (AAF09709); HsIF5, Homo sapiens eIF5A (AAH80196); ScIF5, Saccharomyces cerevisiae eIF5A (AAA34425); MjIF5, Methancaldococcus jannaschii aIF5A (C64453). An overview of a ribbon diagram of the crystal structure of E. coli EF-P (left) and a structural model of N. meningitidis EF-P (right) built by the Phyre2 server [57], using P. aeruginosa EF-P (PDB code: 3OYY) as the template, and visualized by the PyMOL viewer (https://www.pymol.org/). The Lys34 residue in EF-P(Ec) and the corresponding residue (Arg32) in EF-P(Nm) are shown as stick models. Secondary structure assignments (α-helices, 310-helices, and β-sheets) in EF-P(Ec) are represented as α, η, and β, respectively. The α-helices, 310-helices, and β-sheets are colored deep purple, dark olive, and green, respectively. (PDF) [file pone.0147907.s002.pdf]

# Yanagisawa *et al.*, Fig. S2

Domain 1

```
NmEFP MKT--AQELRA-GNVFMVGNDPMVVQKTEYIKGGRSSAKVSMKLKNLLTGAASETIYKADD 58
NgEFP MKT--AQELRA-GNVFMVGNDPMVVQKTEYIKGGRSSAKVSMKLKNLLTGAASETIYKADD 58
BcEFP MKT--AQELRV-GNVVQIGSEAWVIAKAEYNKSGRNSAVVKMKMKNLLSNAGQESVYKADD 58
BpEFP MKT--AQELRV-GNVIMVGKDPLVVQKTEYNKSGRNAAVVKLKFKNLLTGSGSESVYKADE 58
TtEFP MIS--VTDLRP-GTKVKMDGGLWECVEYQHQKLGRGGAKVVAKFKNLETGATVERTFNSGE 58
DrEFP MIS--VTELRN-GTKVQMDGGLWECLDYSHLKMGRGGAKVVTKFRNMESGSIVDRTFNSTE 58
BbEFP MAVVKSSEIEK-GSFLLIKGAPHIVLEREFSKTGRGGAIVRLKLKNLKNKFVIRETLKGAD 60
EcEFP MATYYSNDFRA-GLKIMLDGEPYAVEASEFVKPGKGQAFARVKLRRLLTGTRVEKTFKSTD 60
HsIF5 TFPMQCSALRKNGFVVLK-GRPCKIVEMSTSKTGKHGHAKVHLVGIDIFTGKKYEDICPST 76
ScIF5 TYPMQCSALRKNGFVVIK-GRPCKIVDMSTSKTGKHGHAKVHLVTLDIFTGKKLEDLSPST 77
MjIF5 TKQVNVGSLKV-GQYVMIDGVPCEIVDISVSKPGKHGGAKARVVGIGIFEKVKKEFVAPTS 63
```

Domain 2

```
NmEFP KFDVVILSRKNCTYSYFADPMYVFMD-EEFNQYEIEADNIGDALKFIVDGMED-QCEVTF 116
NgEFP KFDVVILSRKNCTYSYFADPMYVFMD-EEFNQYEIEADNIGDALKFIVDGMED-QCEVTF 116
BcEFP KFEVVMLDRKEVTYSYFADPMYVFMD-ADYNQYEVEAEMMGEALNYLEDGMA---CEVVF 114
BpEFP KFDVVVLERKECTYSYFGDPMYVFMD-EEYNQYEIEADSMGDALNYLEEAMP---VEVVF 114
TtEFP KLEDIYVETRELQYLYPEGEEMVFMDLETYEQFAVPRSRVVG-AEFFKEGMT---ALGDM 114
DrEFP KLQDIYVEGKKMQYLYPDGDDYVFMDMETFDQVHLGKNIVSDAAKFMKENTE---VEVAM 115
BbEFP TAEAIEIYEVSAQYLYKDKDVLVFMDLETYDQVSLDLKESANLQDKVPFLQESEIYSLVT 120
EcEFP SAEGADVVDMNLTYLYNDGEFWHFMNNETFEQLSADAKAIGDNAKWLLDQAE---CIVTL 117
HsIF5 HNMDVPNIKRNDFQLIGIQDGYLSLLQDSGEVREDLRLPEGDLGKEIEQKYDCGEEILIT 136
ScIF5 HNLEVPFVKRSEYQLLDIDDGYLSLMTMDGETKDDVKAPEGELGDSMQAAFDEGKDLMVT 137
MjIF5 SKVEVPIIDRRKGQVLAIMGDMVQIMDL--QTYETLELPIPEGIEGLEPGGEVEYIEAVG 121
```

Domain 3

```
NmEFP YEGNPISVELPTIIVREVEYTEPAVKGDTS-GKVMKTARLVGGTEIQVMSYIENGDKVEI 175
NgEFP YEGNPISVELPTIIVREVEYTEPAVKGDTS-GKVMKTARLVGGTEIQVMSYIENGDKVEI 175
BcEFP YNEKAISVELPTVLVREITYTEPAVKGDTSSGKVLKNAKLATGFELQVPLFCNTGDKIEI 174
BpEFP YDGRAISVELPTILVREITYTEPAVRGDTS-GKVLKPAKINTGFELSVPLFCAIGDKIEI 173
TtEFP YEGQPIKVTPPTVVELKVVDTPPGVRGDTV-SGGSKPATLETGAVVQVPLFVEPGEVIKV 173
DrEFP YGDKALSISLPNQVILKITQTDPGVRGDTV-SGGTKPATLETGAVVQVPLFVEQGTDVKV 174
BbEFP FDNVVIDIKLAPKIAFEVVEVEAAVKGDTV-TNAMKNITLNTGLVVKAPLFINVGDKVLI 179
EcEFP WNGQPISVTPPNFVELEIVDTDPGLKGDTA-GTGGKPATLSTGAVVKVPLFVQIGEVIKV 176
HsIF5 VLSAMTEEAAVAIKAMAK 154
ScIF5 IISAMGEEAAISFKEAPRSD 157
MjIF5 QYKITRVIGGK 132
```

```
NmEFP DTRTGEFRKRA-- 186
NgEFP DTRTGEFRKRA-- 186
BcEFP DTRTNEYRSRA-- 185
BpEFP DTRTNEYRSRVN- 185
TtEFP DTRTGEYVGRA-- 184
DrEFP DTRTGQYLSRA-- 185
BbEFP NSETKEYAERIKN 192
EcEFP DTRSGEYVSRVK- 188
```

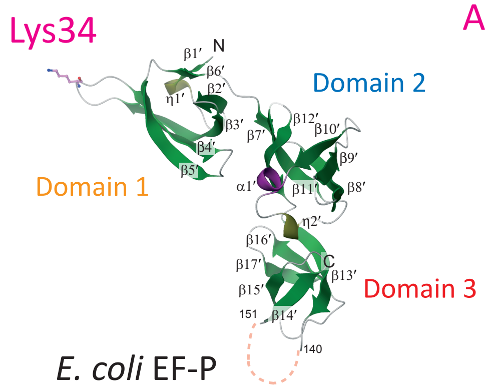


*E. coli* EF-P

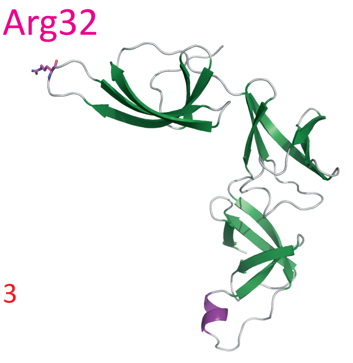


*N. meningitidis* EF-P (model)

**Fig. S2. Structure-based sequence alignment of EF-P and a/eIF5A**

The amino acid sequences were aligned using the program CLUSTAL W (56), and then partly optimized and adjusted manually. Highly conserved residues between EF-P and a/eIF5A are colored red. Highly conserved residues in EF-P are colored orange, and those in a/eIF5A are colored sky blue. The rhamnosylated Arg32 of *N. meningitidis* EF-P is highlighted with a purple asterisk above the sequence alignment. Dashes represent breaks in the actual amino acid sequences of the respective proteins, to allow sequence alignment with *N. meningitidis* EF-P. *Nm*EFP, *N. meningitidis* EF-P (EFV64285); N. *Ec*EFP, *E. coli* EF-P (AAA97046); *Ng*EFP, *Neisseria gonorrhoeae* EF-P (YP_208043); *Bp*EFP, *Bordetella pertussis* EF-P (NP_880680); *Bc*EFP, *Burkholderia cepacia* EF-P (WP_014897640); *Bb*EFP, *Borrelia burgdorferi* EF-P (YP_005806412); *Tt*EFP, *Thermus thermophilus* EF-P (BAD70948); *Dr*EFP, *Deinococcus radiodurans* EF-P (AAF09709); *Hs*IF5, *Homo sapiens* eIF5A (AAH80196); *Sc*IF5, *Saccharomyces cerevisiae* eIF5A (AAA34425); *Mj*IF5, *Methancaldococcus jannaschii* aIF5A (C64453). An overview of a ribbon diagram of the crystal structure of *E. coli* EF-P (left) and a structural model of *N. meningitidis* EF-P (right) built by the Phyre2 server (57), using *P. aeruginosa* EF-P (PDB code: 3OYY) as the template, and visualized by the PyMOL viewer (https://www.pymol.org/). The Lys34 residue in EF-P(*Ec*) and the corresponding residue (Arg32) in EF-P(*Nm*) are shown as stick models. Secondary structure assignments ($\alpha$-helices, $3_{10}$-helices, and $\beta$-sheets) in EF-P(*Ec*) are represented as $\alpha$, $\eta$, and $\beta$, respectively. The $\alpha$-helices, $3_{10}$-helices, and $\beta$-sheets are colored deep purple, dark olive, and green, respectively.
