# Supplementary material for: Neisseria meningitidis Translation Elongation Factor P and Its Active-Site Arginine Residue Are Essential for Cell Viability
Source: PLoS One. 2016 Feb 3;11(2):e0147907. doi: 10.1371/journal.pone.0147907 (PMC4739656; doi:10.1371/journal.pone.0147907)
Supplement: S3 Fig — (A) A crude extract prepared from E. coli cells producing recombinant EF-P(Nm) was incubated with the N. meningitidis crude extract. The extract-treated recombinant EF-P(Nm) was purified, the His6-tag was cleaved with thrombin, and the protein was subjected to MALDI-TOF MS analysis. Lane 1, molecular mass standards; lane 2, crude extract of E. coli cells producing EF-P(Nm) (CE1); lane 3, crude extract of N. meningitidis (CE2); lane 4, molecular mass standards; lane 5, mixture of CE1 and CE2; lane 6, purified recombinant EF-P(Nm). (B, C) The molecular mass of the CE2-treated recombinant EF-P(Nm) (obsd: 21,301.17) is larger by 148.6 Da than that of the recombinant EF-P(Nm) without the incubation with CE2 (calcd: 21,161.02, obsd: 21,152.62). The 149 Da mass increase is comparable to the difference between the endogenous EF-P(Nm) (obsd: 21,034.74) and the recombinant EF-P(Nm) (obsd: 20,887.39). The peaks with masses around 17,000–18,000 Da are presumed to be degradation products. (PDF) [file pone.0147907.s003.pdf]

Yanagisawa *et al.*, Fig. S3

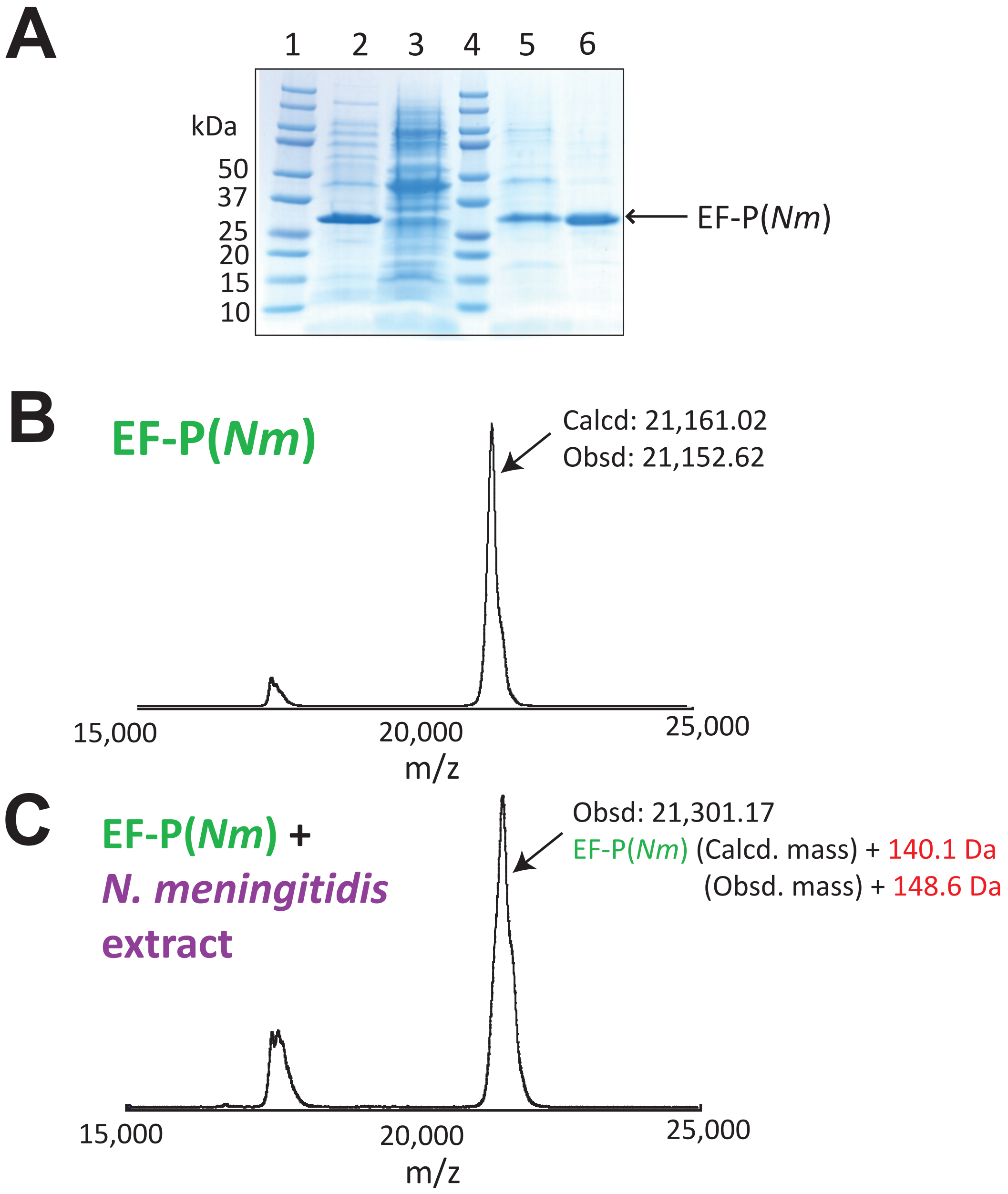

Fig. S3. The activity to modify recombinant EF-P(*Nm*) exists in the crude extract of *N. meningitidis* cells

(A) A crude extract prepared from *E. coli* cells producing recombinant EF-P(*Nm*) was incubated with the *N. meningitidis* crude extract. The extract-treated recombinant EF-P(*Nm*) was purified, the His_6-tag was cleaved with thrombin, and the protein was subjected to MALDI-TOF MS analysis. Lane 1, molecular mass standards; lane 2, crude extract of *E. coli* cells producing EF-P(*Nm*) (CE1); lane 3, crude extract of *N. meningitidis* (CE2); lane 4, molecular mass standards; lane 5, mixture of CE1 and CE2; lane 6, purified recombinant EF-P(*Nm*). (B, C) The molecular mass of the CE2-treated recombinant EF-P(*Nm*) (obsd: 21,301.17) is larger by 148.6 Da than that of the recombinant EF-P(*Nm*) without the incubation with CE2 (calcd: 21,161.02, obsd: 21,152.62). The 149 Da mass increase is comparable to the difference between the endogenous EF-P(*Nm*) (obsd: 21,034.74) and the recombinant EF-P(*Nm*) (obsd: 20,887.39). The peaks with masses around 17,000–18,000 Da are presumed to be degradation products.
