# Supplementary material for: Neisseria meningitidis Translation Elongation Factor P and Its Active-Site Arginine Residue Are Essential for Cell Viability
Source: PLoS One. 2016 Feb 3;11(2):e0147907. doi: 10.1371/journal.pone.0147907 (PMC4739656; doi:10.1371/journal.pone.0147907)
Supplement: S4 Fig — EarP of N. meningitidis HT1125 has 14 different residues from those of N. meningitidis H44/76 and MC58. (PDF) [file pone.0147907.s004.pdf]

Yanagisawa *et al.*, Fig. S4

```
HT1125   1     MNTPPFVCWIFCKVIDNFGDIGVSLRLARVLHRELGWQVHLWTDDVSALRALCPDLPDVP   60
H44/76   1     MNTPPFVCWIFCKVIDNFGDIGVSWRLARVLHRELGWQVHLWTDDVSALRALCPDLPDVP   60
MC58     1     MNTPPFVCWIFCKVIDNFGDIGVSWRLARVLHRELGWQVHLWTDDVSALRALCPDLPDVP   60

HT1125   61    CVHQDIHVRTWHSDAADIDTAPVPDAVIETFACDLPENVLHIIRRHKPLWLNWEYLSAEE   120
H44/76   61    CVHQDIHVRTWHSDAADIDTAPVPDVVIETFACDLPENVLHIIRRHKPLWLNWEYLSAEE   120
MC58     61    CVHQDIHVRTWHSDAADIDTAPVPDVVIETFACDLPENVLHIIRRHKPLWLNWEYLSAEE   120

HT1125   121   SNERLHLMPSPQEGVQKYFWFMGFSEKSGGLIRERDYRDAVRFDTEALRQRLMLPEKNAP   180
H44/76   121   SNERLHLMPSPQEGVQKYFWFMGFSEKSGGLIRERDYCEAVRFDTEALRERLMLPEKNAS   180
MC58     121   SNERLHLMPSPQEGVQKYFWFMGFSEKSGGLIRERDYCEAVRFDTEALRERLMLPEKNAS   180

HT1125   181   EWLLFGYRSDVWAKWLEMWQQAGSPMTLLLAGAQIIDSLKQSGIIPQNALQNDGDVFQTA   240
H44/76   181   EWLLFGYRSDVWAKWLEMWRQAGSPMTLLLAGTQIIDSLKQSGVIPQDALQNDGDVFQTA   240
MC58     181   EWLLFGYRSDVWAKWLEMWRQAGSPMTLLLAGTQIIDSLKQSGVIPQDALQNDGDVFQTA   240

HT1125   241   SVRLVKIPFVPQQDFDQLLHLADCAVIRGEDSFVRAQLAGKPFFWHIYPQDEHVHLDKLH   300
H44/76   241   SVRLVKIPFVPQQDFDQLLHLADCAVIRGEDSFVRAQLAGKPFFWHIYPQDENVHLDKLH   300
MC58     241   SVRLVKIPFVPQQDFDQLLHLADCAVIRGEDSFVRAQLAGKPFFWHIYPQDENVHLDKLH   300

HT1125   301   AFWDKAHGFYTPETASAHRCLSDDLNGGEALSATQRLECWQILQQHQNGWRQGAGAWSRY   360
H44/76   301   AFWDKAHGFYTPETVSAHRRLSDDLNGGEALSATQRLECWQTLQQHQNGWRQGAEDWSRY   360
MC58     301   AFWDKAHGFYTPETVSAHRRLSDDLNGGEALSATQRLECWQTLQQHQNGWRQGAEDWSRY   360

HT1125   361   LFGQPSASEKLAAFVSKHQKIR   382
H44/76   361   LFGQPSAPEKLAAFVSKHQKIR   382
MC58     361   LFGQPSAPEKLAAFVSKHQKIR   382
```

Fig. S4. Sequence alignment of EarP of *N. meningitidis* strains HT1125, H44/76, and MC58

EarP of *N. meningitidis* HT1125 has 14 different residues from those of *N. meningitidis* H44/76 and MC58.
