# Supplementary material for: Neisseria meningitidis Translation Elongation Factor P and Its Active-Site Arginine Residue Are Essential for Cell Viability
Source: PLoS One. 2016 Feb 3;11(2):e0147907. doi: 10.1371/journal.pone.0147907 (PMC4739656; doi:10.1371/journal.pone.0147907)
Supplement: S5 Fig — MALDI-TOF MS spectra of the recombinant EF-P(Nm) purified from E. coli cells producing only EF-P(Nm) (Fig 5A, lane 6) (A), and the recombinant EF-P(Nm) purified from E. coli cells producing both EF-P(Nm) and EarP(Nm) (Fig 5A, lane 7) (B). (PDF) [file pone.0147907.s005.pdf]

Yanagisawa *et al.*, Fig. S5

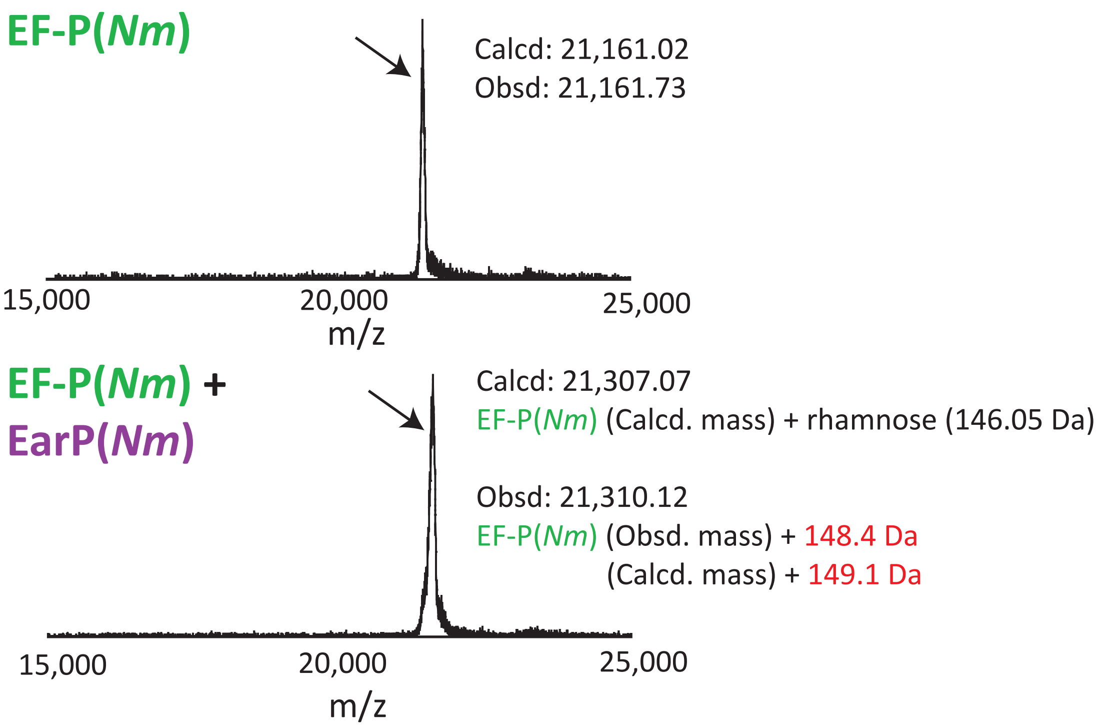

Fig. S5. MS analysis of the modified and unmodified EF-P(*Nm*)

MALDI-TOF MS spectra of the recombinant EF-P(*Nm*) purified from *E. coli* cells producing only EF-P(*Nm*) (Fig. 5A, lane 6) (A), and the recombinant EF-P(*Nm*) purified from *E. coli* cells producing both EF-P(*Nm*) and EarP(*Nm*) (Fig. 5A, lane 7) (B).
