# Supplementary material for: Neisseria meningitidis Translation Elongation Factor P and Its Active-Site Arginine Residue Are Essential for Cell Viability
Source: PLoS One. 2016 Feb 3;11(2):e0147907. doi: 10.1371/journal.pone.0147907 (PMC4739656; doi:10.1371/journal.pone.0147907)
Supplement: S6 Fig — Plasmids pMW119, pMW-NmE (a pMW119-derived plasmid for expressing EF-P(Nm)), pMW-NmED (a pMW119-derived plasmid for expressing EF-P(Nm) and EarP(Nm)), and pMW-EcEGY (a pMW119-derived plasmid for expressing EF-P(Ec), EpmA, and EpmB) were introduced into the E. coli K12 strain BW25113 and the Δefp strain JW4107 (BW25113 Δefp::kan), and the growth of these strains was compared on LB-Amp plates at 37°C. (A) BW25113/pMW119 (B) BW25113/pMW-NmE (C) BW25113/pMW-NmED (D) BW25113/pMW-EcEGY (E) JW4107/pMW119 (F) JW4107/pMW-NmE (G) JW4107/pMW-NmED (H) JW4107/pMW-EcEGY. (I) The growth curves of these cells in LB broth (Miller) medium in the presence of Amp (50 μg/ml) at 37°C are also shown. (PDF) [file pone.0147907.s006.pdf]

Yanagisawa *et al.*, Fig.S6

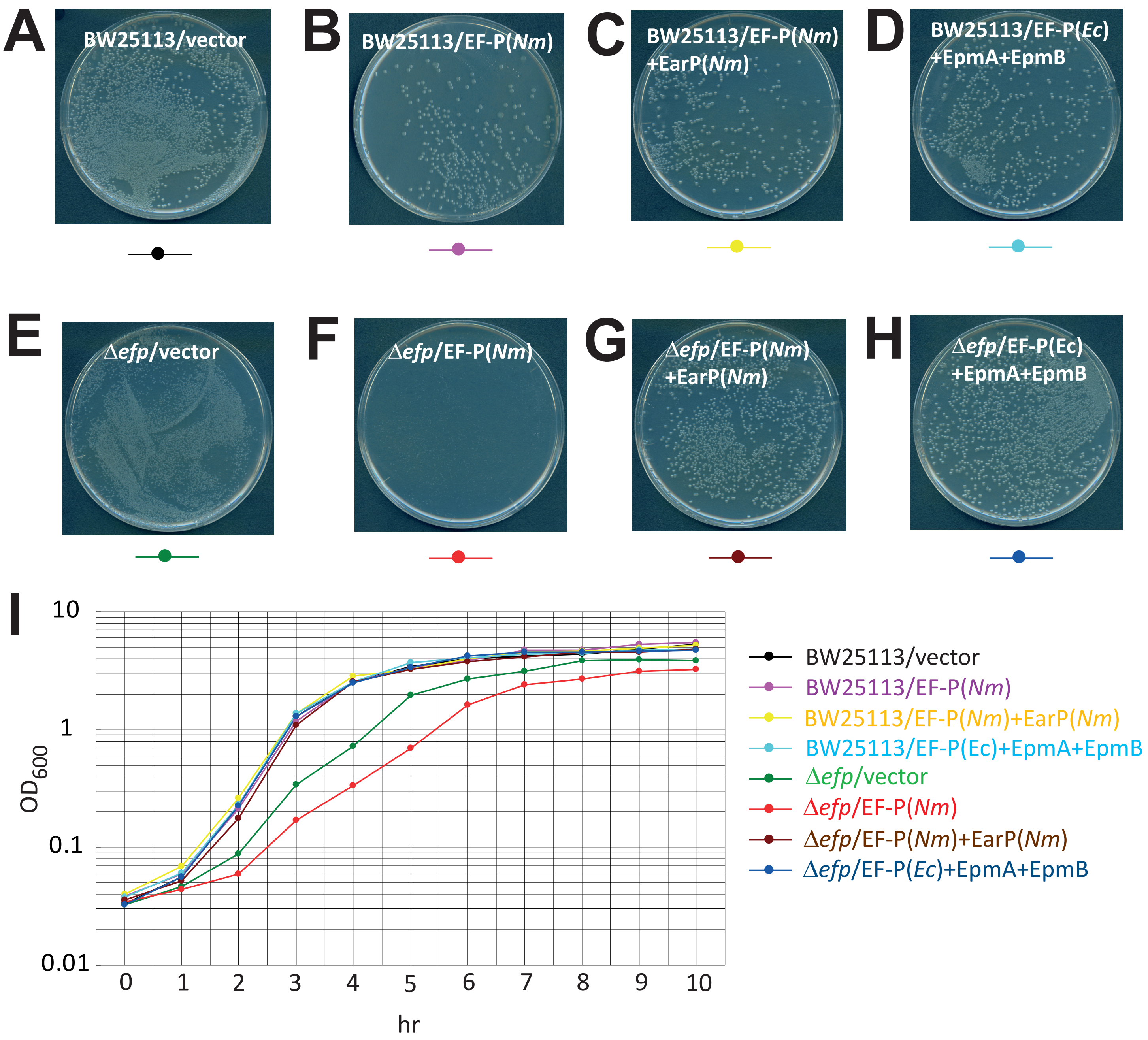

Fig. S6. Complementation analysis of *E. coli efp* null mutant with *N. meningitidis* EF-P

Plasmids pMW119, pMW-*Nm*E (a pMW119-derived plasmid for expressing EF-P(*Nm*)), pMW-*Nm*ED (a pMW119-derived plasmid for expressing EF-P(*Nm*) and EarP(*Nm*)), and pMW-*Ec*EGY (a pMW119-derived plasmid for expressing EF-P(*Ec*), EpmA, and EpmB) were introduced into the *E. coli* K12 strain BW25113 and the Δ*efp* strain JW4107 (BW25113 Δ*efp*::*kan*), and the growth of these strains was compared on LB-Amp plates at 37°C. (A) BW25113/pMW119 (B) BW25113/pMW-*Nm*E (C) BW25113/pMW-*Nm*ED (D) BW25113/pMW-*Ec*EGY (E) JW4107/pMW119 (F) JW4107/pMW-*Nm*E (G) JW4107/pMW-*Nm*ED (H) JW4107/pMW-*Ec*EGY. (I) The growth curves of these cells in LB broth (Miller) medium in the presence of Amp (50 μg/ml) at 37°C are also shown.
