# Supplementary material for: Neisseria meningitidis Translation Elongation Factor P and Its Active-Site Arginine Residue Are Essential for Cell Viability
Source: PLoS One. 2016 Feb 3;11(2):e0147907. doi: 10.1371/journal.pone.0147907 (PMC4739656; doi:10.1371/journal.pone.0147907)
Supplement: S7 Fig — (A) Amino acid sequence of E. coli GntX. Proline stretches in GntX are colored red. (B) Plasmids pMW119, pMW-NmE, pMW-NmED, and pMW-EcEGY were cotransformed with the GntX plasmid into E. coli BW25113 and JW4107 (BW25113 Δefp::kan) cells. Whole-cell proteins were subjected to SDS-PAGE, and analyzed for the production of the full-length GntX protein. Lane 1: molecular mass standards; lane 2: BW25113/pMW119; lane 3: BW25113/pMW-NmE; lane 4: BW25113/pMW-NmED; lane 5: BW25113/pMW-EcEGY; lane 6: JW4107/pMW119; lane 7: JW4107/pMW-NmE; lane 8: JW4107/pMW-NmED; lane 9: JW4107/pMW-EcEGY; lane 10, MagicMark molecular mass standards. (C) The GntX protein was recognized by an anti-His6-antibody. (D) Detection of the expressed N. meningitidis EF-P using an anti-EF-P antibody. (PDF) [file pone.0147907.s007.pdf]

Yanagisawa *et al.*, Fig.S7

A *E. coli* GntX (Calcd.mass 27731)

MRGSHHHHHHTDPALRP
MLTVPGLCWLCRMPLALGHWGICSVCSRATR
TDKTLCPQCGLPATHSHLPCGRCLQKPPPWQ
RLVTVADYAPPLSPLIHQLKFSRRSEIASALSRL
LLLEVLHARRTTGLQLPDRIVSVPLWQRRHWR
RGFNQSDLLCQPLSRWLHCQWDSEAVTRTRA
TATQHFLSARLRKRNLKNAFRLELPVQGRHMV
IVDDVVTTGSTVAEIAQLLLRNGAAAVQVWCLC

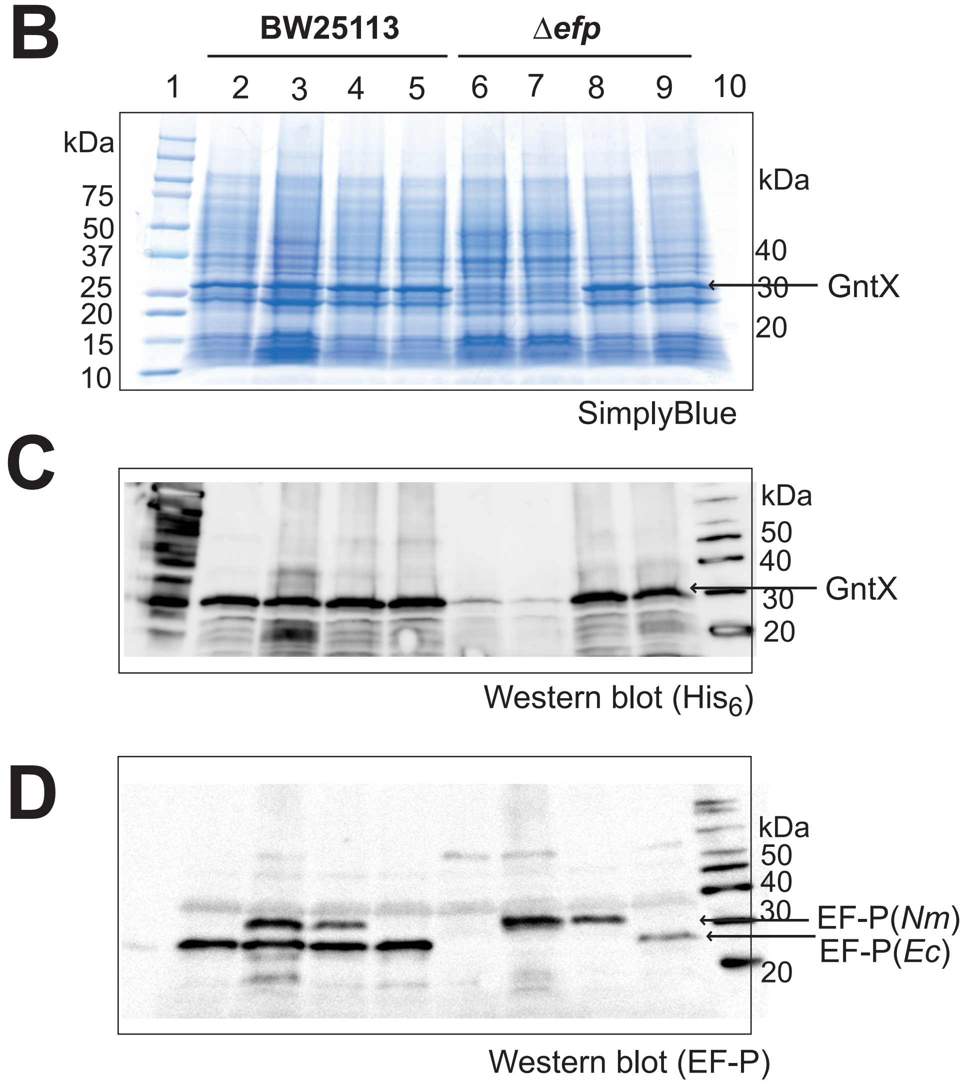

Fig. S7. Restoration of production of the *E. coli* GntX protein, containing proline stretches, in *efp*-deficient cells.

(A) Amino acid sequence of *E. coli* GntX. Proline stretches in GntX are colored red. (B) Plasmids pMW119, pMW-*Nm*E, pMW-*Nm*ED, and pMW-*Ec*EGY were cotransformed with the GntX plasmid into *E. coli* BW25113 and JW4107 (BW25113 Δ*efp*::*kan*) cells. Whole-cell proteins were subjected to SDS-PAGE, and analyzed for the production of the full-length GntX protein. Lane 1: molecular mass standards; lane 2: BW25113/pMW119; lane 3: BW25113/pMW-*Nm*E; lane 4: BW25113/pMW-*Nm*ED; lane 5: BW25113/pMW-*Ec*EGY; lane 6: JW4107/pMW119; lane 7: JW4107/pMW-*Nm*E; lane 8: JW4107/pMW-*Nm*ED; lane 9: JW4107/pMW-*Ec*EGY; lane 10, MagicMark molecular mass standards. (C) The GntX protein was recognized by an anti-His_6-antibody. (D) Detection of the expressed *N. meningitidis* EF-P using an anti-EF-P antibody.
