# Supplementary material for: Neisseria meningitidis Translation Elongation Factor P and Its Active-Site Arginine Residue Are Essential for Cell Viability
Source: PLoS One. 2016 Feb 3;11(2):e0147907. doi: 10.1371/journal.pone.0147907 (PMC4739656; doi:10.1371/journal.pone.0147907)
Supplement: S1 Table — (DOCX) [file pone.0147907.s008.docx]

**Table S1. Strains and plasmids**

| ***E. coli* strain: genotype** | Antibiotic resistance | Source  or references |
| --- | --- | --- |
| BL21-Gold(DE3): E. coli B F^-^ dcm+ Hte ompT hsdS(r_B_- m_B_-) gal λ (DE3) endA Tet^r^ |  | Agilent Technologies Inc. (Santa Clara, USA) |
| BW25113: F^-^, Δ(*araD*-*araB*)567, Δ*lacZ*4787(::*rrnB*-3), λ^-^, rph-1, Δ(*rhaD*-*rhaB*)568, *hsdR*514 |  | Baba *et al*., 2006  (ref. 37) |
| JW4107 (BW25113 Δ*efp::kan*) | Kanamycin  (Km, 50 μg/ml) | Baba *et al*., 2006  (ref. 37) |
| ***N. meningitidis* strain: genotype** |  |  |
| HT1125 wild-type |  | Takahashi *et al.*, 2008a (ref. 48) |
| HT1125 Δ*efp::ermC* /pHT969 | Erythromycin  (Erm, 5 μg/ml)  Chloramphenicol (Cm, 5 μg/ml) | This study |
| HT1125 Δ*earP::spc-*P*lac-efp*  (HT1907) | Spectinomycin (Spc, 75 μg/ml) | This study |
| H44/76 wild-type |  | Takahashi and Watanabe, 2002 (ref. 47) |
| H44/76 Δ*efp::ermC*  (HT1913)/pHT1139 | Chloramphenicol (Cm, 5 μg/ml)  Erythromycin (Erm, 4 μg/ml) | This study |
| H44/76 *earP*-*efp(R32opal)*-*ermC*  (HT1914)/pHT1139 | Chloramphenicol (Cm, 5 μg/ml)  Erythromycin (Erm, 4 μg/ml) | This study |
| **Plasmids** |  |  |
| pGSS33 (IncQ broad-host-range vector) | Chloramphenicol (Cm, 25 μg/ml for *E. coli*; 5 μg/ml for *N. meningitidis*) | Takahashi and Watanabe, 2002 (ref. 47) |
| pHT261 (IncQ broad-host-range vector) | Chloramphenicol (Cm, 25 μg/ml for *E. coli*; 5 μg/ml for *N. meningitidis*) | Takahashi *et al.*, 2008b (ref. 54) |
| pHT969 (pHT261-derived plasmid containing *N. menigitidis* HT1125 *efp* gene) | Chloramphenicol (Cm, 25 μg/ml for *E. coli*; 5 μg/ml for *N. meningitidis*) | This study |
| pHT971 (pHT261-derived plasmid containing *N. menigitidis* HT1125 *efp* gene, in which Arg32 is replaced by Lys32) | Chloramphenicol (Cm, 25 μg/ml for *E. coli*; 5 μg/ml for *N. meningitidis*) | This study |
| pHT972 (pHT261-derived plasmid containing *N. meningitidis* HT1125 *efp* gene, in which Arg32 is replaced by Ala32) | Chloramphenicol (Cm, 25 μg/ml for *E. coli*; 5 μg/ml for *N. meningitidis*) | This study |
| pHT1139 (pGSS33-derived plasmid containing  *lacI^q^* gene and *N. meningitidis* HT1125 *efp* gene with a TTG initiation codon under the *tac* promoter) | Chloramphenicol (Cm, 25 μg/ml for *E. coli*; 5 μg/ml for *N. meningitidis*) | This study |
| pMW119 (plasmid vector) | Ampicillin  (Amp, 50 μg/ml) | Nippon Gene  (Tokyo, Japan) |
| pMW-*Nm*E (pMW119-derived plasmid containing *N. menigitidis* HT1125 *efp* gene and the 0.3-kb 5′- and 0.3-kb 3′-flanking regions) | Ampicillin,  (Amp, 50 μg/ml) | This study |
| pMW-*Nm*E2 (pMW119-derived plasmid containing *N. menigitidis* HT1125 *efp* gene and the 0.6-kb 5′- and 0.5-kb 3′-flanking regions) | Ampicillin,  (Amp, 50 μg/ml) | This study |
| pMW-*Nm*E2-Erm (pMW-NmE2-derived plasmid containing erythromycin resistance gene but without *efp* gene) | Ampicillin,  (Amp, 50 μg/ml) | This study |
| pMW-*Nm*ED (pMW119-derived plasmid containing *N. meningitidis* HT1125 *efp* and *earP* genes) | Ampicillin,  (Amp, 50 μg/ml) | This study |
| pMW-*Ec*EGY (pMW119-derived plasmid containing *E. coli* *efp*, *genX*, and *yjeK* genes) | Ampicillin,  (Amp, 50 μg/ml) | This study |
| pET-*Nm*E1 (pET23-derived plasmid containing *N. menigitidis* H44/76 *efp* gene) | Ampicillin,  (Amp, 50 μg/ml) | This study |
| pET-*Nm*E2 (pET28-derived plasmid containing *N. meningitidis* H44/76 *efp* gene) | Kanamycin  (Km, 50 μg/ml) | This study |
| pET-*Nm*ED (pET28-derived plasmid containing *N. meningitidis* H44/76 *efp* and HT1125 *earP* genes) | Kanamycin  (Km, 50 μg/ml) | This study |
| pET-*Nm*D (pET28-derived plasmid containing *N. meningitidis* HT1125 *earP* gene) | Kanamycin  (Km, 50 μg/ml) | This study |
| pTTQ-*Ptac*-Δ200-TTG-*Nmefp* (pTTQ19-derived plasmid containing *N. meningitidis* HT1125 *efp* gene with a TTG initiation codon under the *tac* promoter) | Ampicillin,  (Amp, 50 μg/ml) | This study |
| pHT1094 (pMW119-derived plasmid containing *N. meningitidis efp*(*R32opal*) | Ampicillin,  (Amp, 50 μg/ml) | This study |
| pHT1095 (pTTQ19-derived plasmid containing 1.1-kb *Bam*HI-*Kpn*I DNA fragment of pMW-*Nm*E) | Ampicillin,  (Amp, 50 μg/ml) | This study |
| pHT1098 (pMW119-derived plasmid containing *N. meningitidis* HT1125 *efp*(*R32opal*) and *ermC* gene) | Ampicillin,  (Amp, 50 μg/ml)  Erythromycin (Erm,150 μg/ml) | This study |
| pHT1088 (pMW119-derived plasmid containing both *earP* (1.2-kb) and *efp* (0.5-kb) genes and their upstream (0.5 kb) and downstream (0.2-kb) regions | Ampicillin,  (Amp, 50 μg/ml)  Erythromycin (Erm, 150 μg/ml) | This study |
| pHT1089 [pMW119-derived plasmid containing *ΔearP::spc*-P*lac-efp* gene (*N. meningitidis* HT1125 *efp* gene under the *lac* promoter)] | Ampicillin,  (Amp, 50 μg/ml)  Spectinomycin (Spc, 75 μg/ml) | This study |
