# Supplementary material for: Neisseria meningitidis Translation Elongation Factor P and Its Active-Site Arginine Residue Are Essential for Cell Viability
Source: PLoS One. 2016 Feb 3;11(2):e0147907. doi: 10.1371/journal.pone.0147907 (PMC4739656; doi:10.1371/journal.pone.0147907)
Supplement: S2 Table — The residues from the pET28-derived His6-tag are shown in blue letters. N. meningitidis HT1125 EF-P has a point mutation in domain III, where the Val173 residue in N. meningitidis H44/76 EF-P is replaced by Leu173, as shown in red letters. The amino acid sequence identities of HT1125 EF-P with those of H44/76 and MC58 are 99.5%. The Arg32 residues are highlighted in purple. (DOCX) [file pone.0147907.s009.docx]

**Table S2. Sequences and calculated masses of *N. meningitidis* EF-P**

| *<* *N. meningitidis* HT1125 EF-P (calcd. mass: 20893.78 Da)>  MKTAQELRAGNVFMVGNDPMVVQKTEYIKGGRSSAKVSMKLKNLLTGAASETIYKADDKFDVVILSRKNCTYSYFADPMYVFMDEEFNQYEIEADNIGDALKFIVDGMEDQCEVTFYEGNPISVELPTIIVREVEYTEPAVKGDTSGKVMKTARLVGGTEIQVMSYIENGDKIEIDTRTGEFRKRA |
| --- |
| *<* *N. meningitidis* H44/76 EF-P (calcd. mass: 20879.75 Da)>  MKTAQELRAGNVFMVGNDPMVVQKTEYIKGGRSSAKVSMKLKNLLTGAASETIYKADDKFDVVILSRKNCTYSYFADPMYVFMDEEFNQYEIEADNIGDALKFIVDGMEDQCEVTFYEGNPISVELPTIIVREVEYTEPAVKGDTSGKVMKTARLVGGTEIQVMSYIENGDKVEIDTRTGEFRKRA |
| *<N. meningitidis* H44/76 EF-P with the His_6_-tag (calcd. mass: 23,043.07 Da)>  MGSSHHHHHHSSGLVPRGSHMKTAQELRAGNVFMVGNDPMVVQKTEYIKGGRSSAKVSMKLKNLLTGAASETIYKADDKFDVVILSRKNCTYSYFADPMYVFMDEEFNQYEIEADNIGDALKFIVDGMEDQCEVTFYEGNPISVELPTIIVREVEYTEPAVKGDTSGKVMKTARLVGGTEIQVMSYIENGDKVEIDTRTGEFRKRA |
| <The His_6_-tag-cleaved *N. meningitidis* H44/76 EF-P (calcd. mass: 21,161.02 Da)>  GSHMKTAQELRAGNVFMVGNDPMVVQKTEYIKGGRSSAKVSMKLKNLLTGAASETIYKADDKFDVVILSRKNCTYSYFADPMYVFMDEEFNQYEIEADNIGDALKFIVDGMEDQCEVTFYEGNPISVELPTIIVREVEYTEPAVKGDTSGKVMKTARLVGGTEIQVMSYIENGDKVEIDTRTGEFRKRA |

The residues from the pET28-derived His_6_-tag are shown in blue letters. *N. meningitidis* HT1125 EF-P has a point mutation in domain III, where the Val173 residue in *N. meningitidis* H44/76 EF-P is replaced by Leu173, as shown in red letters. The amino acid sequence identities of HT1125 EF-P with those of H44/76 and MC58 are 99.5%. The Arg32 residues are highlighted in purple.
