# Supplementary material for: Neisseria meningitidis Translation Elongation Factor P and Its Active-Site Arginine Residue Are Essential for Cell Viability
Source: PLoS One. 2016 Feb 3;11(2):e0147907. doi: 10.1371/journal.pone.0147907 (PMC4739656; doi:10.1371/journal.pone.0147907)
Supplement: S3 Table — All of the proteins encoded in the N. meningitidis MC58 genome were searched for the presence of proline stretches. Proline–proline–proline–proline (PPPP), proline–proline–proline (PPP), aspartic acid–proline–proline (DPP), proline–proline–tryptophan (PPW), proline–proline–aspartic acid (PPD), alanine–proline–proline (APP), proline–proline–asparagine (PPN), and proline–proline–glycine (PPG) residues are colored red. (DOCX) [file pone.0147907.s010.docx]

**Table S3. Proline stretch-containing proteins encoded in the *N. meningitidis* MC58 genome**

| Proline stretches | Annotations | Putative functions | *E. coli* homologues  (essential or non-essential gene in *E. coli*) |
| --- | --- | --- | --- |
| LPPPPR, DPPY | NMB1061 (ComJ) | 23S rRNA m(6) A2030 methyltransferase | +  *rlmJ/yhiR* (non-essential) |
| QPPPPV | NMB1067 (FtsK-2) | cell division protein | +  *ftsK* (essential) |
| FPPPPI | NMB0980 (PntA) | NAD(P) transhydrogenase, subunit α) | +  *pntA* (non-essential) |
| LPPPV | NMB0898 | unknown | — |
| CPPPE | NMB0117 (Smg) | unknown | +  *smg* (non-essential) |
| TPPPR | NMB0118 (TopA) | topoisomerase I | +  *topA* (essential) |
| LPPPN | NMB0174 (ValS) | valyl-tRNA synthetase | +  *valS* (essential) |
| YPPPK | NMB0439 (NnrS) | unknown | — |
| SPPPD | NMB0475 | unknown | — |
| TPPPA, LPPG | NMB0551 (PriA) | primosome assembly protein | +  *priA* (non-essential) |
| TPPPR, APPG, APPH | NMB0564 (NqrF) | Na(+)-translocating NADH-quinone  reductase subunit F | + (partial homology)  *hmp* |
| GPPPL | NMB0580 (NosL) | protein disulfide isomerase | — |
| LPPPK | NMB0657 | unknown | — |
| APPPA | NMB0785 (RecB) | exodeoxyribonuclease V beta subunit | +  *recB* (non-essential) |
| APPPF | NMB0794 (VanZ) | unknown | — |
| VPPPH | NMB0809 (RsuA) | 23S rRNA pseudouridine(2604) synthase | +  *rsuA* (non-essential) |
| VPPPT | NMB0846 (RfaG) | LPS biosynthesis protein-related protein | + (partial homology)  *wcaL* (non-essential) |
| SPPPS | NMB1116 | DNA polymerase alpha subunit B N-terminal | — |
| APPPD | NMB1128 | unknown | — |
| APPPD | NMB1166 | unknown | — |
| KPPPD | NMB1211 | unknown | — |
| TPPPQ | NMB1225 | unknown | — |
| APPPK | NMB1314 (FtsK-1) | cell division protein | +  *ftsK* (essential) |
| KPPPR | NMB1317 | unknown | — |
| EPPPT | NMB1331 (UvrB) | excinuclease ABC subunit B | +  *uvrB* (non-essential) |
| APPPT | NMB1342 (AceF) | pyruvate dehydrogenase E2 component (dihydrolipoamide acetyltransferase) | +  *aceF* (non-essential) |
| IPPPD, APPPE, IPPW | NMB1443 (DnaX) | DNA polymerase III subunits gamma and tau | +  *dnaX* (essential) |
| HPPPV | NMB1508 (alkPPc) | alkaline phosphatase homologue | — |
| LPPPK, APPL, GPPG | NMB1519 (DipZ) | thiol:disulfide interchange protein DsbD | +  *zipZ* (non-essential) |
| CPPPQ | NMB1529 | unknown | — |
| QPPPL | NMB1535 | unknown | — |
| TPPPA | NMB1541 (IbpB) | lactoferrin-binding protein | — |
| LPPPD | NMB1620 | murein tetrapeptide carboxypeptidase; LD-carboxypeptidase A | +  *ldcA* (non-essential) |
| SPPPN | NMB1671 (PqiB) | paraquat-inducible protein B | +  *pqiB* (non-essential) |
| VPPPI | NMB1715 (MtrD) | multiple transferable resistance system protein | +  *acrB* (non-essential) |
| EPPPV, APPD | NMB1735 (RelA) | GTP pyrophosphokinase | +  *relA* (non-essential) |
| QPPPH | NMB1759 (Fic) | unknown | — |
| TPPPT | NMB1776 | unknown | — |
| SPPPG, LPPG | NMB1788 (RecG) | ATP-dependent DNA helicase | +  *recG* (non-essential) |
| FPPPE | NMB1830 (Gph) | phosphoglycolate phosphatase | +  *gph* (non-essential) |
| LPPPQ, APPF | NMB1871 | unknown | + (partial homology)  *yeaZ* |
| CPPPA | NMB1898 (MlpJ) | lipoprotein | — |
| NPPPT | NMB1907 (YidC) | inner membrane protein translocase component | +  *yidC* (essential) |
| APPPN | NMB1969 | serotype-1-specific antigen | — |
| LPPPS | NMB1986 | unknown | — |
| TPPPQ | NMB2035 | unknown | — |
| YPPPG | NMB2105 (MafB) | unknown | — |
| DPPF | NMB0122 | methyltransferase | +  *yhhF* (non-essential) |
| DPPV | NMB0277 (MviN) | virulence factor | +  *mviN* (essential) |
| DPPR | NMB0349 (YadB) | glutamyl-Q tRNA(Asp) synthetase | +  *yadB* (non-essential) |
| DPPL | NMB0570 | unknown | — |
| DPPI | NMB0650 | unknown | — |
| DPPI | NMB0651 | unknown | — |
| DPPI | NMB0660 | unknown | — |
| DPPY | NMB0727 | N-6 adenine-specific DNA methylase | +  *hsdM* (non-essential) |
| DPPL | NMB0927 (Pip) | proline iminopeptidase | — |
| DPPA, APPD | NMB1016 | unknown | — |
| DPPS | NMB1367 | methyltransferase | +  *yccW* (non-essential) |
| DPPF | NMB1559 (GshB) | glutathione synthetase | +  *gshB* (non-essential) |
| DPPR | NMB1679 (TrmA) | tRNA(uracil-5-)-  methyltransferase | +  *trmA* (non-essential) |
| DPPL, TPPN | NMB1797 | penicillin-binding protein 3 | +  *ftsI* (essential) |
| DPPT | NMB2019 (CoaD) | phosphopantetheine adenylyltransferase | +  *coaD* (essential) |
| TPPD | NMB0005 (ArsC) | arsenate reductase | +  *arsC* (non-essential) |
| APPD | NMB0279 | unknown | — |
| APPD | NMB0437 | unknown | — |
| TPPD | NMB0599 | unknown |  |
| FPPD, IPPN | NMB0990 | unknown | — |
| TPPD, APPE | NMB1327 | unknown | +  *ybeQ* (non-essential) |
| IPPD | NMB1504 (ScpA) | segregation and condensation protein A | — |
| MPPD | NMB1536 (SecA) | preprotein translocase subunit | +  *secA* (essential) |
| KPPD | NMB1694 | unknown | +  *ubiD* (non-essential) |
| LPPD | NMB1792 | sensor histidine kinase | + (partial homology)  *basR* (non-essential) |
| LPPD | NMB1827 (DnaE) | DNA polymerase III subunit alpha | +  *dnaE* (essential) |
| TPPD | NMB1954 | unknown | — |
| LPPD | NMB2026 | ABC transporter permease | +  *thiP* (non-essential) |
| APPD | NMB2051 (PetC) | cytochrome C | — |
| APPA | NMB0071 (CtrA) | capsule polysaccharide export outer membrane protein | — |
| APPV | NMB0111 (Fmt) | methionyl-tRNA formyltransferase | +  *fmt* (essential) |
| APPV | NMB0116 (DprA) | DNA processing protein | +  *dprA* (non-essential) |
| APPI | NMB0168 (RpoA) | DNA-directed RNA polymerase subunit alpha | +  *rpoA* (essential) |
| APPV | NMB0214 (PrlC) | oligopeptidase A | +  *prlC* (non-essential) |
| APPK | NMB0244 (NuoD) | NADH dehydrogenase subunit D | +  *nuoC* (non-essential) |
| APPA | NMB0295 (Ffh) | signal recognition particle protein | +  *ffh* (essential) |
| APPA | NMB0338 | unknown | — |
| APPQ | NMB0395 | unknown | — |
| APPH | NMB0466 (AspS) | aspartyl-tRNA synthetase | +  *aspS* (essential) |
| APPQ | NMB0529 | unknown |  |
| APPL | NMB0590 (TrmD) | tRNA(guanine-N(1)-)-methyl  transferase | +  *trmD* (essential) |
| APPK | NMB0617 (Rho) | transcription termination factor | +  *rho* (essential) |
| APPA | NMB0621 | unknown | — |
| APPS | NMB0673 | unknown | — |
| APPS, APPS | NMB0700 (Iga) | IgA-specific serine endopeptidase | — |
| APPG | NMB0836 (ClpA) | ATP-dependent Clp protease ATP-binding subunit | +  *clpA* (non-essential) |
| APPS | NMB0842 (RecJ) | single-stranded-DNA-specific exonuclease |  |
| APPS | NMB0938 | unknown | — |
| APPR | NMB1095 | unknown | — |
| APPT | NMB1266 | zinc uptake regulation protein | +  ORF_f191 (non-essential) |
| APPF | NMB1306 | unknown | +  *yhcM* (non-essential) |
| APPL | NMB1349 | unknown | — |
| APPF | NMB1359 | CDP-6-deoxy-delta-3,4-  glucose reductase | + (partial homology)  *fre* (non-essential) |
| APPT | NMB1364 | NH(3)-dependent NAD synthetase | +  *nadE* (essential) |
| APPK | NMB1377 (LldD) | L-lactate dehydrogenase | +  *lldD* (non-essential) |
| APPS | NMB1441 | O-methyltransferase | — |
| APPF | NMB1445 (RecA) | recombinase A | +  *recA* (non-essential) |
| APPG | NMB1472 (ClpB) | ATP-dependent Clp protease ATP-binding subunit | +  *clpB* (non-essential) |
| APPF | NMB1514 (DnaQ-2) | DNA polymerase III subunit epsilon | +  *dnaQ* (non-essential) |
| APPH | NMB1531 | unknown | — |
| APPT, VPPG | NMB1572 (AcnB) | bifunctional aconitate hydratase 2/2-methylisocitrate dehydratase | +  *acnB* (non-essential) |
| APPY | NMB1622 (NorB) | nitric oxide reductase | — |
| APPA | NMB1670 | paraquat-inducible protein A | +  *pqiA* (non-essential) |
| APPK | NMB1703 (FabF-2) | 3-oxoacyl-ACP synthase | +  *fabF* (non-essential) |
| APPY | NMB1784 | unknown | — |
| APPE | NMB1813 (AroK) | shikimate kinase | +  *aroK* (non-essential) |
| APPV | NMB1814 (AroB) | 3-dehydroquinate synthase | +  *aroB* (non-essential) |
| APPL, LPPN | NMB1817 (RibD) | riboflavin biosynthesis protein | +  *ribD* (essential) |
| APPA | NMB1818 | lipopolysaccharide biosynthesis protein | — |
| APPL, APPR | NMB1837 | unknown | — |
| APPE | NMB1897 (LeuS) | leucyl-tRNA synthetase | +  *leuS* (essential) |
| APPE | NMB1919 | ABC transporter ATP-binding protein | +  *msbA* (essential) |
| APPL | NMB1998 | serine-type peptidase | — |
| APPT | NMB2036 (TruA) | tRNA pseudouridine synthase A | +  *truA* (non-essential) |
| APPK | NMB2048 (LigA-2) | DNA ligase | — |
| APPM | NMB2096 (YojH) | malate:quinone oxidoreductase | +  *mqo* (non-essential) |
| APPN | NMB2124 | unknown | — |
| APPS | NMB2133 | serine/threonine transporter SstT | +  *sstT* (non-essential) |
| APPL | NMB2136 | peptide transporter | +  *dtpD* (non-essential) |
| IPPN | NMB0268 | RNA methyltransferase | +  *trmL* (non-essential) |
| LPPN | NMB0638 (GalU) | UTP-glucose-1-phosphate uridylyltransferase | +  *galU* (non-essential) |
| LPPN | NMB0676 | unknown | — |
| SPPN, TPPN | NMB0792 | transporter | +  *pyrD* (non-essential) |
| TPPN | NMB0819 | unknown | — |
| IPPN | NMB0849 (Dcd) | deoxycytidine triphosphate deaminase | +  *dcd* (non-essential) |
| EPPN | NMB0871 (PanC) | pantoate-beta-alanine ligase | +  *panC* (non-essential) |
| YPPN, LPPN | NMB1066 | unknown | — |
| YPPN | NMB1096 | unknown | — |
| LPPN | NMB1326 (UvrC) | excinuclease ABC subunit C | +  *uvrC* (non-essential) |
| LPPN | NMB1605 (ParC) | DNA topoisomerase IV subunit A | +  *parC* (essential) |
| NPPN | NMB1698 | acyltransferase | — |
| FPPN | NMB1786 | unknown | — |
| QPPN | NMB1855 (CarB) | carbamoyl phosphate synthase large subunit | +  *carB* (non-essential) |
| LPPN | NMB1967 | AraC family transcriptional regulator | + (partial homology)  *rob* (non-essential) |
| TPPN | NMB1999 (MgtE) | magnesium transporter | +  *yhiD* (non-essential) |
| FPPG | NMB0035 | unknown | +  *ycdO* (non-essential) |
| MPPG | NMB0064 (GalE) | UDP-glucose 4-epimerase | +  *galE* (non-essential) |
| GPPG | NMB0405 (ComM) | putative ATP-dependent protease | +  *yifB* (non-essential) |
| RPPG | NMB0545 | unknown | — |
| RPPG | NMB0605 | histone deacetylases and histone-like deacetylases | — |
| LPPG | NMB0806 | unknown | — |
| MPPG | NMB0882 | unknown | — |
| VPPG | NMB0954 (GltA) | type II citrate synthase | +  *gltA* (non-essential) |
| LPPG | NMB998 | oxidoreductase | + (partial homology)  *glcD* |
| VPPG | NMB1039 | unknown | — |
| GPPG | NMB1231 (Lon) | ATP-dependent protease La | +  *lon* (non-essential) |
| GPPG | NMB1243 (RuvB) | Holliday junction DNA helicase | +  *ruvB* (non-essential) |
| GPPG | NMB1258 (RarA) | recombination factor protein | +  *rarA* (non-essential) |
| LPPG | NMB1281 (Mfd) | transcription-repair coupling factor | +  *mfd* (non-essential) |
| LPPG | NMB1444 | DNA-binding protein, putative nucleoid-associated protein | +  *ybaB* (non-essential) |
| LPPG | NMB1467 (GppA) | exopolyphosphatase | +  *ppx* (non-essential) |
| LPPG | NMB1565 | unknown | — |
| VPPG | NMB1582 (HisC) | histidinol-phosphate aminotransferase | +  *hisC* (non-essential) |
| CPPG | NMB1613 (FumB) | fumarate hydratase | +  *fumB* (non-essential) |
| LPPG | NMB1649 (DsbB) | disulfide bond formation protein | +  *dsbB* (non-essential) |
| CPPG | NMB1680 (AroC) | chorismate synthase | +  *aroC* (non-essential) |
| NPPG | NMB1700 | unknown | — |
| NPPG | NMB1782 | unknown | — |
| FPPG | NMB1794 (CitM) | citrate transporter | — |
| VPPG | NMB1831 (IspH) | 4-hydroxy-3-methylbut-2-enyl diphosphate reductase | +  *ispH* (essential) |
| LPPG | NMB1846 | antiporter inner membrane protein | +  *mrp* (non-essential) |
| EPPG | NMB1934 (AtpD) | F-type H+-transporting ATPase subunit beta | +  *atpD* (non-essential) |

All of the proteins encoded in the *N. meningitidis* MC58 genome were searched for the presence of proline stretches. Proline–proline–proline–proline (PPPP), proline–proline–proline (PPP), aspartic acid–proline–proline (DPP), proline–proline–tryptophan (PPW), proline–proline–aspartic acid (PPD), alanine–proline–proline (APP), proline–proline–asparagine (PPN), and proline–proline–glycine (PPG) residues are colored red.
