# Supplementary material for: Neisseria meningitidis Translation Elongation Factor P and Its Active-Site Arginine Residue Are Essential for Cell Viability
Source: PLoS One. 2016 Feb 3;11(2):e0147907. doi: 10.1371/journal.pone.0147907 (PMC4739656; doi:10.1371/journal.pone.0147907)
Supplement: S4 Table — Pathogenic bacteria containing EF-P(Arg32) and rhamnosyl modification enzymes (EarP homologues) are colored red, and those containing EF-P(Lys32/33/34), EpmA, and EpmB are colored blue. B. burgdorferi containing EF-P(Arg32) but lacking a EarP homologue is colored orange. (DOCX) [file pone.0147907.s011.docx]

**Table S4. EF-P modification enzymes in pathogenic bacteria**

| **Pathogenic bacteria** | **Phylum/**  **Class** | **Conserved Lys or Arg in EF-P** | **EpmA**  **conserved?** | **EpmB**  **conserved?** | **EarP**  **conserved?** |
| --- | --- | --- | --- | --- | --- |
| *Actinomadura madurae* | Actinobacteria/  Actinobacteria | Lys32 | no | no | no |
| *Actinomyces pyogenes* | Actinobacteria/  Actinobacteria | Lys32 | no | no | no |
| *Aeromonas hydrophila* | Proteobacteria/  γ-proteobacteria | Arg32 | no | no | Yes(402a.a.) |
| *Arcanobacterium haemolyticum* | Actinobacteria/  Actinobacteria | Lys32 | no | no | no |
| *Bacillus cereus* | Firmicutes/  Bacilli | Lys32 | no | no | no |
| *Bacteroides fragilis* | Bacteroidetes/  Bacteroidia | Lys32 | no | no | no |
| *Bartonella clarridgeiae* | Proteobacteria/  α-proteobacteria | Lys33 | no | no | no |
| *Bordetella avium* | Proteobacteria/  β-proteobacteria | Arg32 | no | no | Yes (358a.a.) |
| *Bordetella bronchiseptica* | Proteobacteria/  β-proteobacteria | Arg32 | no | no | Yes (370a.a.) |
| *Bordetella pertussis* | Proteobacteria/  β-proteobacteria | Arg32 | no | no | Yes (370a.a.) |
| *Bordetella petrii* | Proteobacteria/  β-proteobacteria | Arg32 | no | no | Yes (369a.a.) |
| *Borrelia burgdorferi* | Spirochaetes/  Spirochaetes | Arg32 | no | no | no |
| *Brucella suis* | Proteobacteria/  α-proteobacteria | Lys33 | no | no | no |
| *Burkholdelia cepacia* | Proteobacteria/  β-proteobacteria | Arg32 | no | no | Yes (397a.a.) |
| *Burkholdelia pseudomallei* | Proteobacteria/  β-proteobacteria | Arg32 | no | no | Yes (406a.a.) |
| *Campylobacter jejuni* | Proteobacteria/  ε-proteobacteria | Lys34 | no | no | no |
| *Chlamydia trachomatis* | Chlamydiae/  Chlamydiae | Lys34 | no | no | no |
| *Chlamydphila pneumoniae* | Chlamydiae/  Chlamydiae | Lys34 | no | no | no |
| *Citrobacter freundii* | Proteobacteria/  γ-proteobacteria | Lys34 | Yes (325 a.a.) | Yes(342 a.a.) | no |
| *Citrobacter rodentium* | Proteobacteria/  γ-proteobacteria | Lys34 | Yes (325 a.a.) | Yes(342 a.a.) | no |
| *Clostridium botulinum* | Firmicutes/  Clostridia | Lys32 | no | no | no |
| *Clostridium tetani* | Firmicutes/  Clostridia | Lys32 | no | no | no |
| *Corynebacterium diphtheriae* | Actinobacteria/  Actinobacteria | Lys32 | no | no | no |
| *Coxiella burnetii* | Proteobacteria/  γ-proteobacteria | Lys34 | Yes (321a.a.) | Yes (342a.a.) | no |
| *Enterobacter aerogenes* | Proteobacteria/  γ-proteobacteria | Lys34 | Yes (325a.a.) | Yes (343a.a.) | no |
| *Enterococcus faecalis* | Firmicutes/  Bacilli | Lys32 | no | no | no |
| *Escherichia coli* | Proteobacteria/  γ-proteobacteria | Lys34 | Yes (325a.a.) | Yes (342a.a.) | no |
| *Francisella philomiradia* | Proteobacteria/  γ-proteobacteria | Lys34 | Yes (307a.a.) | Yes (328a.a.) | no |
| *Haemophilus influenzae* | Proteobacteria/  γ-proteobacteria | Lys34 | Yes (323a.a.) | Yes (338a.a.) | no |
| *Helicobacter pylori* | Proteobacteria/  ε-proteobacteria | Lys33 | no | no | no |
| *Kingella kingae* | Proteobacteria/  β-proteobacteria | Arg32 | no | no | Yes (377a.a.) |
| *Klebsiella pneumoniae* | Proteobacteria/  γ-proteobacteria | Lys34 | Yes (325a.a.) | Yes (342a.a.) | no |
| *Legionella pneumophila* | Proteobacteria/  γ-proteobacteria | Lys34 | Yes (317a.a.) | Yes (326a.a.) | no |
| *Leptospira interrogans* | Spirochaetes/spirochaetes | Lys33 | Yes (324a.a.) | Yes (365a.a.) | no |
| *Listeria monocytogenes* | Firmicutes/  Bacilli | Lys32 | no | no | no |
| *Moraxella catarralis* | Proteobacteria/  γ-proteobacteria | Lys34 | Yes (324a.a.) | Yes (356a.a.) | no |
| *Mycobacterium tuberculosis* | Actinobacteria/  Actinobacteria | Lys32 | no | no | no |
| *Mycoplasma pneumoniae* | Tenericutes/  Mollicutes | Lys33 | no | no | no |
| *Neisseria gonorrhoeae* | Proteobacteria/  β-proteobacteria | Arg32 | no | no | Yes (383a.a.) |
| *Neisseria meningitidis* | Proteobacteria/  β-proteobacteria | Arg32 | no | no | Yes (382a.a.) |
| *Nocardia brasiliensis* | Actinobacteria/  Actinobacteria | Lys32 | no | no | no |
| *Pasteurella multocida* | Proteobacteria/  γ-proteobacteria | Lys34 | Yes (323a.a.) | Yes (337a.a.) | no |
| *Proteus mirabilis* | Proteobacteria/  γ-proteobacteria | Lys34 | Yes (325a.a.) | Yes (342a.a.) | no |
| *Pseudomonas aeruginosa* | Proteobacteria/  γ-proteobacteria | Arg32 | no | no | Yes (376a.a.) |
| *Ralstonia solanacearum* | Proteobacteria/  β-proteobacteria | Arg32 | no | no | Yes (396a.a) |
| *Rickettsia prowazekii* | Proteobacteria/  α-proteobacteria | Lys34 | no | no | no |
| *Salmonella enterica* | Proteobacteria/  γ-proteobacteria | Lys34 | Yes (325a.a.) | Yes (342a.a.) | no |
| *Serratia marcescens* | Proteobacteria/  γ-proteobacteria | Lys34 | Yes (325a.a.) | Yes (342a.a.) | no |
| *Shigella flexneri* | Proteobacteria/  γ-proteobacteria | Lys34 | Yes (325a.a.) | Yes (342a.a.) | no |
| *Staphylococcus aureus* | Firmicutes/  Bacilli | Lys32 | no | no | no |
| *Streptococcus pneumoniae* | Firmicutes/  Bacilli | Lys33 | no | no | no |
| *Treponema pallidum* | Spirochaetes/  Spirochaetes | Lys32 | Yes (351a.a.) | Yes (353a.a.) | no |
| *Ureaplasma urealyticum* | Tenericutes/  Mollicutes | Lys32 | no | no | no |
| *Vibrio cholerae* | Proteobacteria/  γ-proteobacteria | Lys34 | Yes (324a.a.) | Yes (342a.a.) | no |
| *Yersinia pestis* | Proteobacteria/  γ-proteobacteria | Lys34 | Yes (325a.a.) | Yes (342a.a.) | no |

Pathogenic bacteria containing EF-P(Arg32) and rhamnosyl modification enzymes (EarP homologues) are colored red, and those containing EF-P(Lys32/33/34), EpmA, and EpmB are colored blue. *B. burgdorferi* containing EF-P(Arg32) but lacking a EarP homologue is colored orange.
